# *OsASR5* enhances drought tolerance through a stomatal closure pathway associated with ABA and H_2_O_2_ signalling in rice

**DOI:** 10.1111/pbi.12601

**Published:** 2016-11-11

**Authors:** Jinjie Li, Yang Li, Zhigang Yin, Jihong Jiang, Minghui Zhang, Xiao Guo, Zhujia Ye, Yan Zhao, Haiyan Xiong, Zhanying Zhang, Yujie Shao, Conghui Jiang, Hongliang Zhang, Gynheung An, Nam‐Chon Paek, Jauhar Ali, Zichao Li

**Affiliations:** ^1^ Key Lab of Crop Heterosis and Utilization of Ministry of Education and Beijing Key Lab of Crop Genetic Improvement China Agricultural University Beijing People's Republic of China; ^2^ Department of Plant Systems Biotech and Crop Biotech Institute Kyung Hee University Yongin Korea; ^3^ Department of Plant Science, Plant Genomics and Breeding Institute Research Institute for Agriculture and Life Sciences Seoul National University Seoul Korea; ^4^ International Rice Research Institute Metro Manila Philippines

**Keywords:** Drought, *Oryza sativa*, *OsASR5*, water content, ABA, stomata

## Abstract

Drought is one of the major abiotic stresses that directly implicate plant growth and crop productivity. Although many genes in response to drought stress have been identified, genetic improvement to drought resistance especially in food crops is showing relatively slow progress worldwide. Here, we reported the isolation of *abscisic acid*,* stress* and *ripening* (*ASR*) genes from upland rice variety, IRAT109 (*Oryza sativa* L. ssp. *japonica*), and demonstrated that overexpression of *OsASR5* enhanced osmotic tolerance in *Escherichia coli* and drought tolerance in *Arabidopsis* and rice by regulating leaf water status under drought stress conditions. Moreover, overexpression of *OsASR5* in rice increased endogenous ABA level and showed hypersensitive to exogenous ABA treatment at both germination and postgermination stages. The production of H_2_O_2_, a second messenger for the induction of stomatal closure in response to ABA, was activated in overexpression plants under drought stress conditions, consequently, increased stomatal closure and decreased stomatal conductance. In contrast, the loss‐of‐function mutant, *osasr5*, showed sensitivity to drought stress with lower relative water content under drought stress conditions. Further studies demonstrated that OsASR5 functioned as chaperone‐like protein and interacted with stress‐related HSP40 and 2OG‐Fe (II) oxygenase domain containing proteins in yeast and plants. Taken together, we suggest that *OsASR5* plays multiple roles in response to drought stress by regulating ABA biosynthesis, promoting stomatal closure, as well as acting as chaperone‐like protein that possibly prevents drought stress‐related proteins from inactivation.

## Introduction

Drought is a major environmental stress affecting plant growth and reducing crop productivity. Due to the water shortage and inadequate rainfall in the rice‐growing season, improving drought resistance becomes especially important for stabilizing rice productivity and production. However, drought resistance is a complex trait that involves a series of physiological, morphological, cellular and molecular adaptive pathways (Nguyen *et al*., 1997; Umezawa *et al*., 2006; Valliyodan and Nguyen, 2006), resulting in a quite slow progress in the genetic improvement of drought resistance worldwide.

Multiple strategies are adapted by plants in response to drought stress; among them, drought avoidance and drought tolerance are the two major mechanisms for improving drought resistance (Luo, 2010; Price *et al*., 2002). Drought avoidance assists plants maintaining tissue water potential by deep root and reducing water loss, especially through promoting stomatal closure (Hu and Xiong, 2014). Upon drought stress, abscisic acid (ABA), a key plant hormone, increases dramatically, which in turn leads to a number of molecular and cellular responses, among which the best known are inducing stress‐related genes and triggering stomatal closure (Daszkowska‐Golec and Szarejko, 2013; Lee and Luan, 2012; Ye *et al*., 2012). Significant research findings over the last 10 years have shown that ABA stimulates H_2_O_2_ generation mainly by NADPH oxidase in guard cells, and the generated H_2_O_2_ plays a vital role as essential signal molecules that mediate ABA‐induced stomatal closure by activating plasma membrane calcium channels (Kwak *et al*., 2003; Mustilli *et al*., 2002; Pei *et al*., 2000; Wang and Song, 2008; Zhang *et al*., 2001). Recently, H_2_O_2_‐induced stomatal closure through ABA‐independent pathway was reported in rice. A zinc finger transcription factor, *DST*, negatively regulates H_2_O_2_‐induced stomatal closure by the direct modulation of genes related to H_2_O_2_ scavenging (Huang *et al*., 2009). A rice homologue of SRO, *OsSRO1c,* increased stomatal closure by the regulation of H_2_O_2_ homeostasis possibly through down‐regulation of *DST* (You *et al*., 2013). So far, the genes that regulate stomatal movement through ABA‐dependent and H_2_O_2_‐mediated pathway in crops have not been identified, and the mechanism of stomata‐regulated drought tolerance in crops is largely unknown.

In most species, *abscisic acid*,* stress* and *ripening* (*ASR*) genes belong to a small gene family that is characterized by the presence of an ABA/WDS domain, and have been identified from monocot to dicot; nevertheless, they do not present in *Arabidopsis* (Gonzalez and Iusem, 2014). *ASR* genes were found to express in various organs and growth stages among different species, and responsive to ABA and various abiotic stresses, including drought, cold and salt stresses (Cakir *et al*., 2003; Chen *et al*., 2011; Henry *et al*., 2011; Hu *et al*., 2013; Huang *et al*., 2000; Joo *et al*., 2013; Kalifa *et al*., 2004; Maskin *et al*., 2001; Perez‐Diaz *et al*., 2014; Philippe *et al*., 2010; Saumonneau *et al*., 2012). Although these genes were discovered two decades earlier and were reported in response to diverse abiotic stresses, till date we lack the complete understanding of the exact molecular functions and physiological roles under drought stress.

Yeast one‐hybrid experiments revealed that the grape (*Vitis vinifera*) ASR ortholog named VvMSA binds to the promoter of a hexose transporter gene *VvHT1* (Cakir *et al*., 2003). By the yeast two‐hybrid approach, a protein partner of VvMSA was isolated and characterized as a DREB transcription factor (Saumonneau *et al*., 2008). Likewise, tobacco (*Nicotiana tabacum*) ASR ortholog named NtTIP1 interacts with a tobacco bZIP transcription factor *in vivo,* and they possibly function in flower development and stress response (Hwan *et al*., 2012). Until recently, genome‐wide chromatin immunoprecipitation data identified that rice OsASR5 binds to the promoter of the putative targets genes, including an ABC transporter required for Al tolerance (Arenhart *et al*., 2014). Similarly, the targets of tomato ASR1 were reported to be genes involving in cell wall synthesis and remodelling as well as water transporter like aquaporins (Ricardi *et al*., 2014). Interestingly, tomato, plantain and lily ASR proteins were reported to perform a chaperone‐like activity that protects reporter enzymes from denaturation induced by freezing or heat *in vitro* (Dai *et al*., 2011; Hsu *et al*., 2011; Konrad and Bar‐Zvi, 2008). Moreover, several studies on the heterologous and homologous expression of *ASR* genes in plant species were reported for functional characterization of *ASR* genes. Overexpression of the *ASR* gene from plantain (*Musa paradisiaca*;* MpASR*) and lily (*Lilium longiflorum*;* LLA23*) in *Arabidopsis* enhanced osmotic, cold and freezing tolerances possibly by acting as osmoprotectant, respectively (Dai *et al*., 2011; Hsu *et al*., 2011). Transgenic tobacco plants overexpressing the *ASR* gene from tomato (*Solanum lycopersicum*;* ASR1*) or *Salicornia brachiata* (*SbASR‐1*) exhibited improved tolerance to osmotic stress (Jha *et al*., 2012; Kalifa *et al*., 2004) and from wheat (*Triticum aestivum*;* TaASR1*) showed enhanced tolerance to water stress (Hu *et al*., 2013). The ZmASR1 protein influences branched‐chain amino acid biosynthesis and transgenic maize (*Zea mays*) plants overexpression of *ZmASR1* maintained kernel yield under water‐limited conditions (Virlouvet *et al*., 2011). Overexpression of *OsASR1* or *OsASR3* in transgenic rice plants also resulted in enhanced tolerances to cold and drought stresses in terms of photosynthetic efficiency (Joo *et al*., 2013; Kim *et al*., 2009). It appears that the exact functions of the ASR proteins are still baffling, as the possible roles of the *ASR* genes could not be simply deduced by sequence homology with other known proteins (Virlouvet *et al*., 2011).

Upland rice (UR) has been evolved as ‘drought‐resistant type’ derived from natural and artificial selection under drought stress conditions, while lowland rice (LR) is ‘drought‐sensitive type’ in rice; thus, identification and elucidating the function of drought‐responsive genes from UR will promote our understanding of drought tolerance mechanism in rice. To gain new insight into ASR functions in response to drought stress, we characterized the *ASR* gene family from UR variety, IRAT109 (*O. sativa* L. ssp. *japonica*). Three drought‐responsive *ASR* genes, *OsASR3*,* OsASR5* and *OsASR6*, were identified from UR, and using *OsASR5* overexpression plants and the loss‐of‐function mutant, the function and molecular mechanism of *OsASR5* in drought tolerance were characterized and discussed, respectively.

## Results

### Expression profile of *ASR* genes in UR and LR

Genes preferentially expressed in UR under drought stress conditions were the probable candidate genes to improve drought tolerance. For that reason, the expression changes in the *ASR* genes in response to drought were analysed between UR variety, IRAT109, and LR variety, Nipponbare (*O. sativa* L. ssp. *japonica*). Rice contains six *ASR* paralogous genes (Philippe *et al*., 2010); among them, *OsASR3* was up‐regulated in IRAT109, and *OsASR5* and *OsASR6* were induced and up‐regulated by drought in IRAT109 relative to Nipponbare (Figure S1). To further study the functions of the *ASR* genes in response to abiotic stress in rice, we currently focused on the characterization of *OsASR5*.

**Figure 1:**
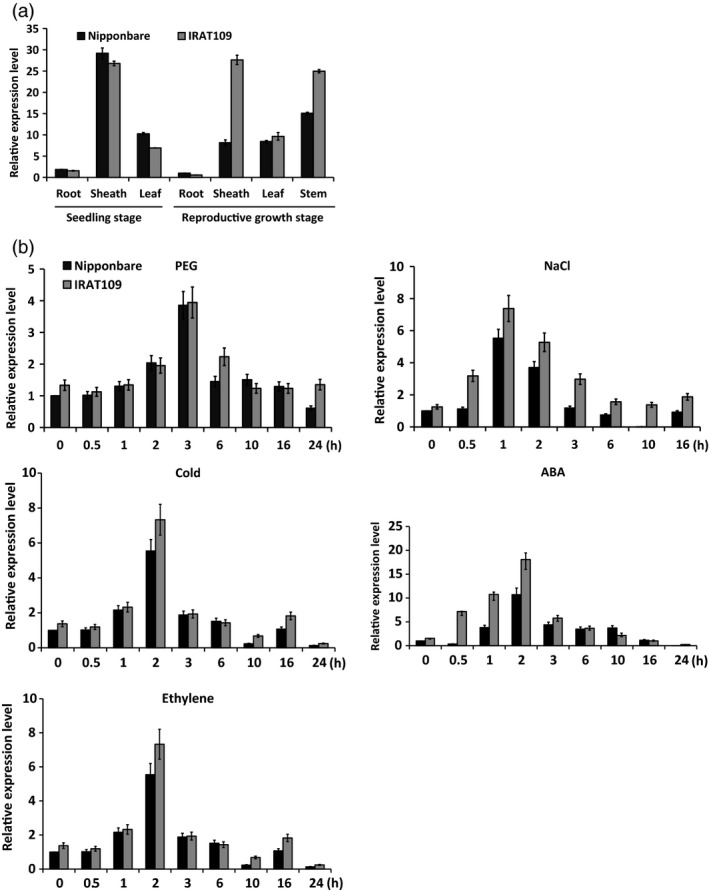
Expression analysis of the *OsASR5* gene. (a) Real‐time PCR analysis of the expression level of *OsASR5* in different tissues of LR variety, Nipponbare, and UR variety, IRAT109. (b) Stress‐inducible expression of *OsASR5* under PEG, NaCl, cold, ABA and ethylene treatments. Error bars indicate standard error (SE) based on three replicates.

To investigate whether the tissue‐specific expression of *OsASR5* is different between the two varieties, the expression patterns of *OsASR5* in various organs during seedling and productive stages were analysed by quantitative real‐time PCR (qRT‐PCR). As shown in Figure 1a, *OsASR5* was expressed in various organs at seedling and reproductive stages, interestingly, highly expressed in the sheath and stem tissues of IRAT109 as compared to Nipponbare during reproductive stage. The temporal and spatial expression pattern of *OsASR5* was further investigated by transforming Nipponbare with a fusion gene of *Pro*_*OsASR5*_:*OsASR5‐*GFP. The GFP signal was observed in pistil, stamen, glume, guard cell, leaf, root, sheath and in vascular bundles (Figure S2).

To speculate the function of *OsASR5,* the transcript levels of *OsASR5* in response to polyethylene glycol (PEG), high salinity, cold, ABA and ethylene were analysed in the leaf tissues. The *OsASR5* transcript was induced rapidly by PEG, NaCl, cold, ABA and ethylene for 1–3 h after treatments both in IRAT109 and in Nipponbare; interestingly, the expression levels of *OsASR5* in IRAT109 were much higher than those in Nipponbare (Figure 1b). For instance, there was a significant increase in the *OsASR5* transcripts in 1–2 h after ABA treatment in both varieties; however, the transcript levels of *OsASR5* showed 1.5‐ to 2.0‐folds in IRAT109 as compared with Nipponbare. These data suggest that *OsASR5* was responsive to multiple abiotic stresses preferentially in UR variety.

### Expression of *OsASR5* enhances osmotic and drought tolerance in *E. coli* and Arabidopsis

**Figure 2:**
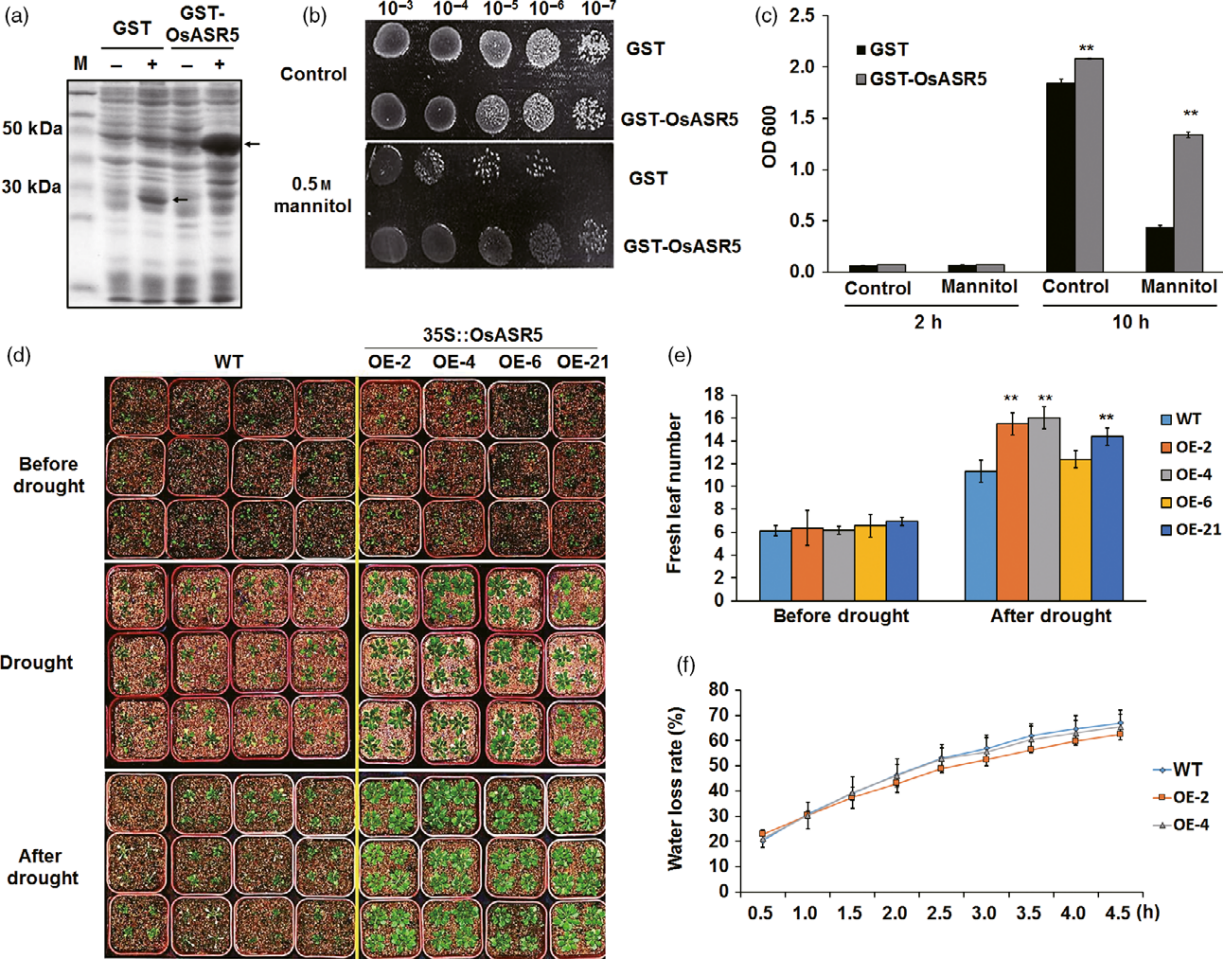
Enhanced osmotic and drought tolerance in *E. coli* and *Arabidopsis*. (a) Isopropylb‐D‐thiogalactopyranoside (IPTG)‐inducible expression of GST and GST‐OsASR5 fusion proteins. GST and GST‐OsASR5 were not (−) or were (+) induced by IPTG. Arrows indicate expression proteins. (b) Growth analysis of cells spotted on LB agar plate supplemented with 0.5 m mannitol. (c) Growth analysis of cells cultured in liquid medium supplemented with 0.5 m mannitol (*n *=* *3). Cell growth densities were measured at 600 nm at the indicated time points. (d) Drought stress tolerance assay of *OsASR5* overexpression *Arabidopsis* transgenic lines and WT by stopping irrigation for 3 weeks and recovery with rewatering for 4 days. (e) Fresh leaf numbers of *OsASR5* overexpression *Arabidopsis* transgenic lines and WT before and after drought stress (*n *=* *3, four plants in each repeat). (f) Water loss rate in the detached leaves of *OsASR5* overexpression *Arabidopsis* transgenic lines and WT under normal conditions (*n *=* *3, 12 leaves in each repeat). Data are mean ± SE. ** indicates significant difference at *P* < 0.01 probability.

To examine the potential role of *OsASR5* in protecting cells from osmotic stress, heterologous expression of *OsASR5* in *E. coli* (BL21) was carried out. Cells transformed with the empty vector were used as a control (Figure 2a). The growth of the cells transformed either with empty vector or with recombinant plasmid showed nonsignificant differences on fresh LB media. On solid media containing 0.5 m mannitol, the transformants expressing GST‐OsASR5 fusion protein showed higher growth rate than those expressed GST protein only (Figure 2b). On liquid media with 0.5 m mannitol, the growth rate of the transformants expressing GST‐OsASR5 fusion protein was threefold higher than the control after incubation for 10 h (Figure 2c). These results clearly indicate that the heterologous expression of OsASR5 protein increased *E. coli* tolerance to osmotic stress.

To examine whether overexpression of *OsASR5* in *Arabidopsis* would increase the tolerance of transgenic lines to drought stress, ten T_3_ transgenic lines were obtained and four of them with highest transcript levels of *OsASR5* were used to verify the function of *OsASR5* (Figure S3). There existed no developmental differences between overexpressed and wild‐type (WT) plants under the normal conditions. However, under drought stress conditions, the transgenic plants gave more green leaves with higher leaf area as compared with WT plants that showed a significant inhibited growth. Moreover, all of the transgenic plants showed the complete recovery after rewatering, while only half of the WT recovered, and water loss rates of detached leaves from transgenic plants were lower than those from WT. These results indicate that heterologous overexpression of *OsASR5* in *Arabidopsis* enhances drought tolerance, suggesting that *OsASR5* is functional in dicots.

### Overexpression of *OsASR5* significantly enhances osmotic and drought tolerance in rice

**Figure 3:**
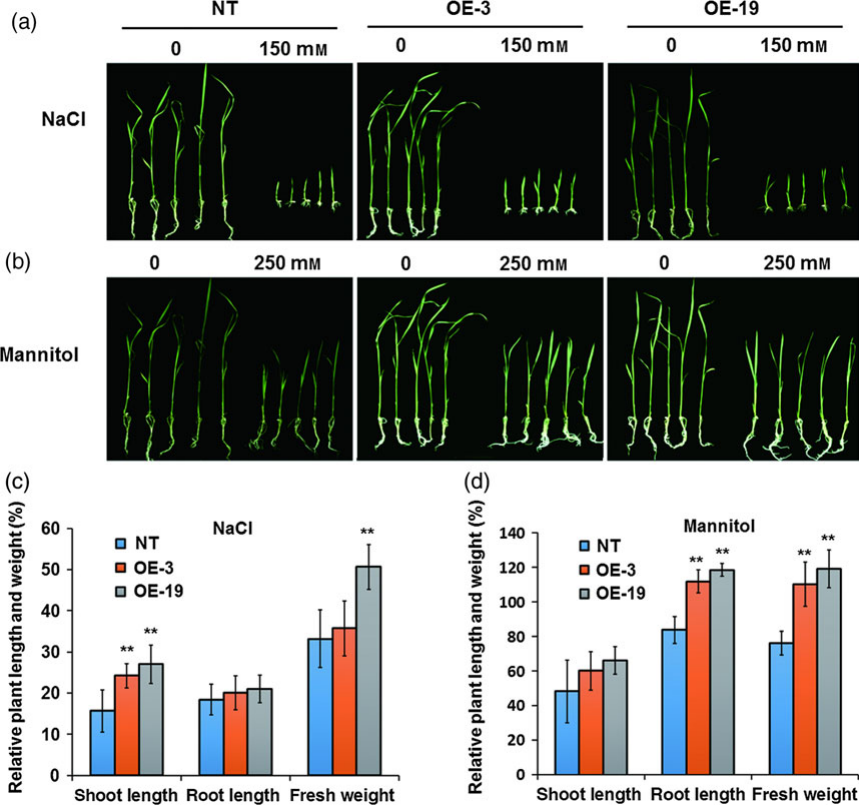
Increased osmotic tolerance of *OsASR5* overexpression plants. (a, b) Growth performance of *OsASR5* overexpression and NT seedlings under high salinity and mannitol treatments at the seventh d after transplanting, respectively (*n *=* *3, five plants in each repeat). (c, d) The relative plant length and fresh weight of *OsASR5* overexpression and NT seedlings corresponding to a, b, respectively. Data are mean ± SE. ** indicates significant difference at *P* < 0.01 probability.

To directly investigate the function of *OsASR5* in response to osmotic and drought stress in rice, seven transgenic lines with overexpressing *OsASR5* were obtained. Of them, two lines (OE‐3 and OE‐19) with highest transcription levels of *OsASR5* were selected to verify the function of *OsASR5* (Figure S4). The performances of *OsASR5* overexpression lines under high osmotic stress caused by adding high salinity or mannitol were examined. The growth of the *OsASR5* overexpression seedlings was less inhibited (Figure 3a,b), exhibiting higher relative shoot growth and relative shoot fresh weight than those of the nontransgenic (NT) seedlings (Figure 3c,d). These results indicate that overexpressing *OsASR5* in rice could enhance the tolerance of overexpression lines to osmotic stress.

**Figure 4:**
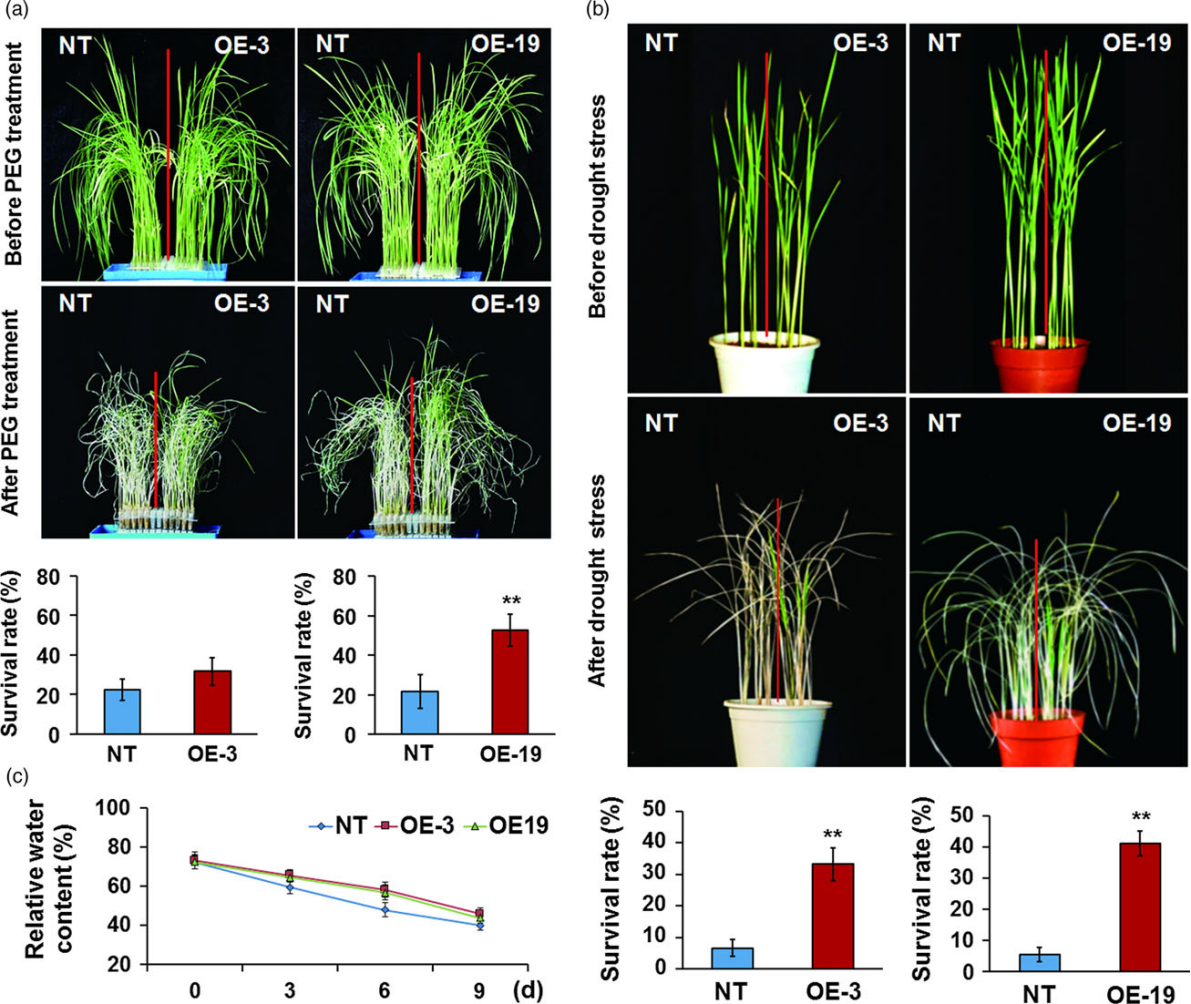
Enhanced drought tolerance of *OsASR5* overexpression plants. (a) Physiological dehydration stress tolerance assay of *OsASR5* overexpression and NT plants under 15% PEG6000 treatment. Survival rates of *OsASR5* overexpression and NT plants after dehydration stress were examined (*n *=* *3, 15 plants in each repeat). (b) Drought stress tolerance assay of *OsASR5* overexpression and NT plants by stopping irrigation for 1 week and recovery with rewatering for 2 weeks. Survival rates of *OsASR5* overexpression and NT plants after drought stress were examined (*n *=* *3, nine plants in each repeat). (c) Relative water content of *OsASR5* overexpression and NT plants under 15% PEG6000 treatment at the indicated time points (*n *=* *3). Data are mean ± SE. ** indicates significant difference at *P* < 0.01 probability.

Three‐week‐old seedlings grown in liquid medium were treated with PEG to create physiological dehydration stress conditions; after recovery was performed, *OsASR5* overexpression lines showed a stronger growth recovery phenotype than that of the NT plants. Almost 31.6%–52.5% of *OsASR5* overexpression plants survived, while only 21.6%–22.5% of the NT survived under this treatment (Figure 4a). Furthermore, the *OsASR5* overexpression and NT plants were planted in the soil and well watered at the tillering stage. There were no developmental differences between *OsASR5* overexpression and NT plants when normal irrigation was performed. However, after 1 week of stopping irrigation and 2 weeks of recovery, the *OsASR5* overexpression lines showed a distinct recovery rate from that of the NT plants. Almost 33.3%–41.1% of *OsASR5* overexpression plants survived, whereas only 5.5%–6.6% of the NT plants survived this treatment (Figure 4b). Therefore, it is evidence that overexpression of *OsASR5* results in increased tolerance to drought stress in rice.

Because relative water content (RWC) is one of the most important traits to detect drought tolerance, the RWC in the leaves of *OsASR5* overexpression and NT plants was measured during drought stress. High RWC in the leaves of *OsASR5* overexpression plants was observed as compared with that of NT (Figure 4c), suggesting that *OsASR5* possibly plays an important role in reducing water evaporation especially under drought stress conditions. To further evaluate the physiological and biochemical changes in *OsASR5* overexpression plants, the contents of free proline and soluble carbohydrates in *OsASR5* overexpression and NT plants were measured following drought stress. The contents of proline and sugar in both *OsASR5* overexpression and NT plants rose continuously during drought treatment, whereas no significant differences were observed between *OsASR5* overexpression and NT plants (Figure S5), suggesting that overexpression of *OsASR5* does not regulate the accumulation of proline and sugar in transgenic plants.

### Overexpression of *OsASR5* increases stomatal closure

**Figure 5:**
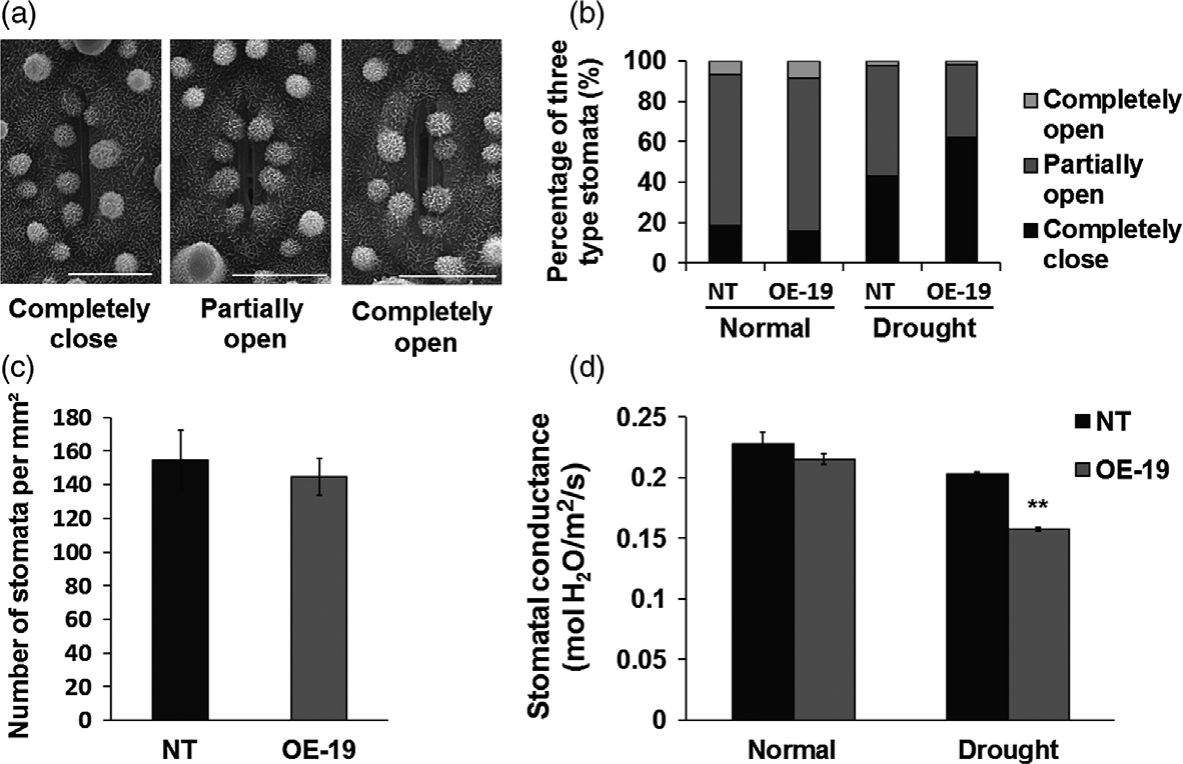
Overexpression of *OsASR5* increasing stomatal closure. (a) Scanning electron microscopy images of three levels of stomatal apertures. Bar, 5 μm. (b) The percentage of three levels of stomatal apertures in the leaves of *OsASR5* overexpression and NT plants under normal and drought stress conditions (*n *=* *300 stomata for NT under normal conditions; *n *=* *248 stomata for OE‐19 under normal conditions; *n *=* *322 stomata for NT under drought stress; *n *=* *272 stomata for OE‐19 under drought stress). (c) Stomatal density of the middle leaves of *OsASR5* overexpression and NT plants (*n *=* *3). Three random scopes were used in each repeat. (d) Stomatal conductance of *OsASR5* overexpression and NT plants (*n *=* *3). Data are mean ± SE. ** indicates significant difference at *P* < 0.01 probability.

As water loss is mainly occurred through stomatal opening in plants, the reduced water loss in the *OsASR5* overexpression plants prompted us to investigate stomatal aperture. The leaf stomatal apertures of *OsASR5* overexpression and NT plants were observed by using scanning electron microscopy. As shown in Figure 5a,b, the percentages of completely closure, completely open and partially open stomata in the *OsASR5* overexpression plants were not obviously different as compared with the NT plants under normal conditions. However, under drought stress conditions, 62.0% of stomata completely closed in the *OsASR5* overexpression plants, while only 43.1% completely closed in the NT plants; in contrast, only 36.7% partially opened in the *OsASR5* overexpression plants, but 54.4% partially opened in the NT plants, whereas nonsignificant differences in the percentage of completely open stomata were observed. Nonsignificant differences were observed for the stomatal density between overexpression and NT plants (Figure 5c). Moreover, the stomatal conductance was obviously decreased in *OsASR5* overexpression plants as compared with the NT plants under drought stress conditions (Figure 5d). These results clearly demonstrate that overexpressing *OsASR5* possibly affects the stomatal movements especially under drought stress conditions.

### Overexpression of *OsASR5* increased endogenous ABA level and sensitivity to exogenous ABA

**Figure 6:**
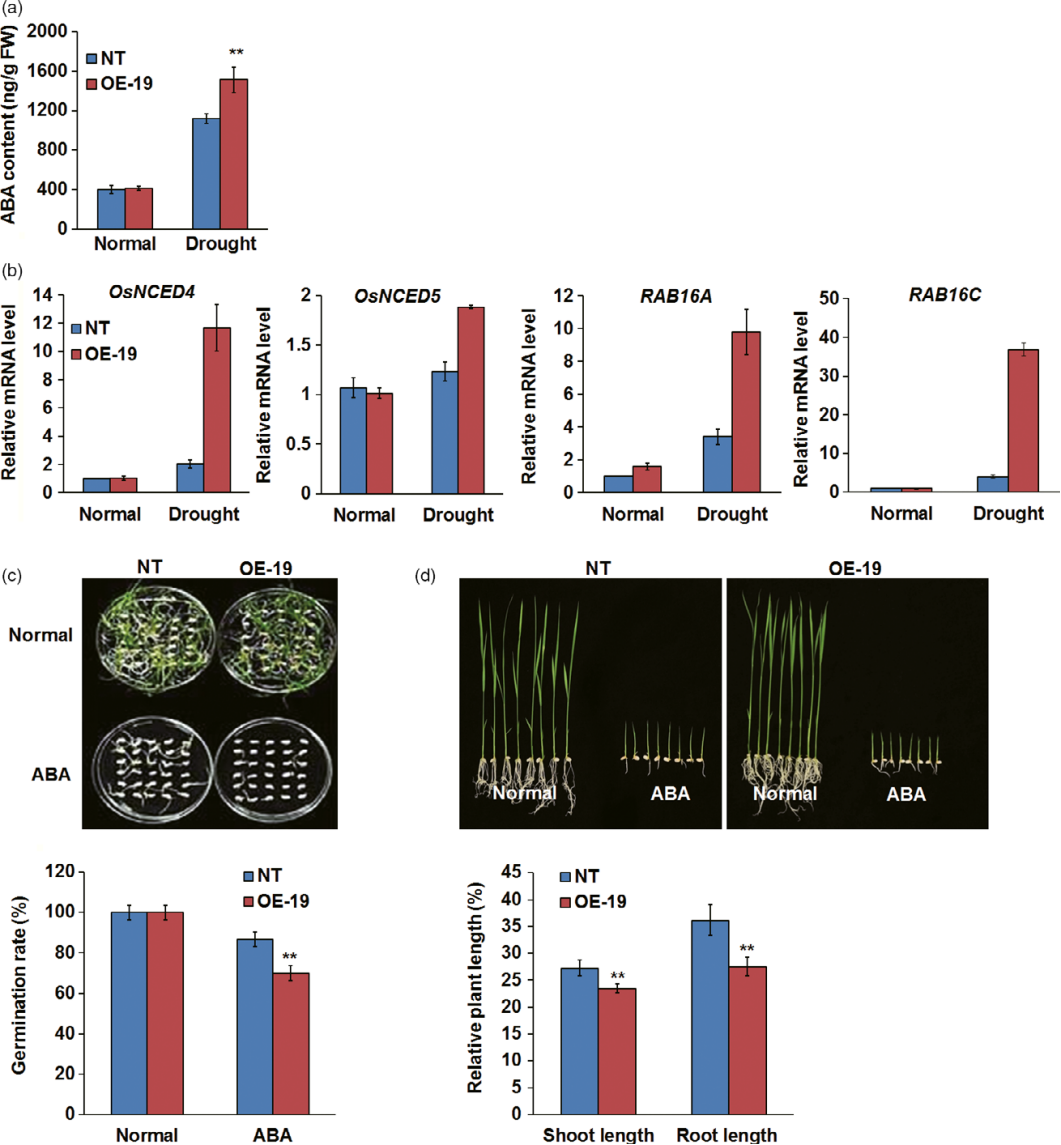
ABA accumulation and sensitivity of *OsASR5* overexpression plants. (a) ABA contents of *OsASR5* overexpression and NT plants under normal and drought stress conditions (*n *=* *3). (b) Real‐time PCR analysis of the expression of ABA biosynthesis and responsive genes under normal and drought stress conditions (*n *=* *3). (c) Germination rates of *OsASR5* overexpression and NT seeds under ABA treatment (*n *=* *3, 30 seeds in each repeat). (d) Growth performance and relative plant length of *OsASR5* overexpression and NT seedlings under ABA treatment (*n *=* *3, five plants in each repeat). Data are mean ± SE. ** indicates significant difference at *P* < 0.01 probability.

Because ABA can induce stomatal closure and consequently decrease water loss, the endogenous ABA levels were measured in the leaves of *OsASR5* overexpression and NT plants under normal and drought stress conditions. The result showed that the endogenous ABA levels in both *OsASR5* overexpression and NT seedlings were clearly increased by drought stress; interestingly, the level of ABA was much higher in *OsASR5* overexpression seedlings (1513 ng/g fresh weight) than that in NT seedlings (1120 ng/g fresh weight), whereas no obvious difference was observed under normal conditions (Figure 6a). We hypothesized that *OsASR5* may be involved in regulating ABA biosynthesis in drought stress. To confirm this, the transcript levels of ABA biosynthesis and responsive genes were analysed. As shown in Figure 6b, the expression levels of *OsNCED4* and *OsNCED5*,* RAB16A* and *RAB16C* were highly up‐regulated by drought stress in *OsASR5* overexpression seedlings as compared with NT seedlings, whereas the expression levels of these genes showed nonsignificant differences between *OsASR5* overexpression and NT seedlings under normal conditions. These results indicate that *OsASR5* may play an important role in drought‐induced ABA biosynthesis through up‐regulation of ABA biosynthesis genes, such as *OsNCED4* and *OsNCED5*, and regulating ABA‐responsive genes, such as *RAB16A* and *RAB16C*.

As the expression of *OsASR5* was induced by ABA, we speculate that *OsASR5* may play a positive role in ABA signalling in rice. To confirm this hypothesis, the exogenous ABA sensitivities of *OsASR5* overexpression lines were investigated at germination and postgermination stages. As shown in Figure 6c, no obvious difference in germination rate was observed between overexpression and NT plants in the normal medium, whereas the germination rates of overexpression lines were significantly lower than those of the NT plants in the medium with 2.5 μm ABA at the end of treatment. Similarly, the relative shoot length and root length of the *OsASR5* overexpression plants were significantly shorter than those of the NT plants, while nonsignificant differences were observed for the growth rate between *OsASR5* overexpression and NT seedlings in the normal medium at the postgermination stage (Figure 6d). These results demonstrate that overexpressing *OsASR5* increased exogenous ABA sensitivity at both germination and postgermination stages, indicating that *OsASR5* may be a positive regulator of ABA signalling in rice.

### *OsASR5* modulates H_2_O_2_ homeostasis in drought stress

**Figure 7:**
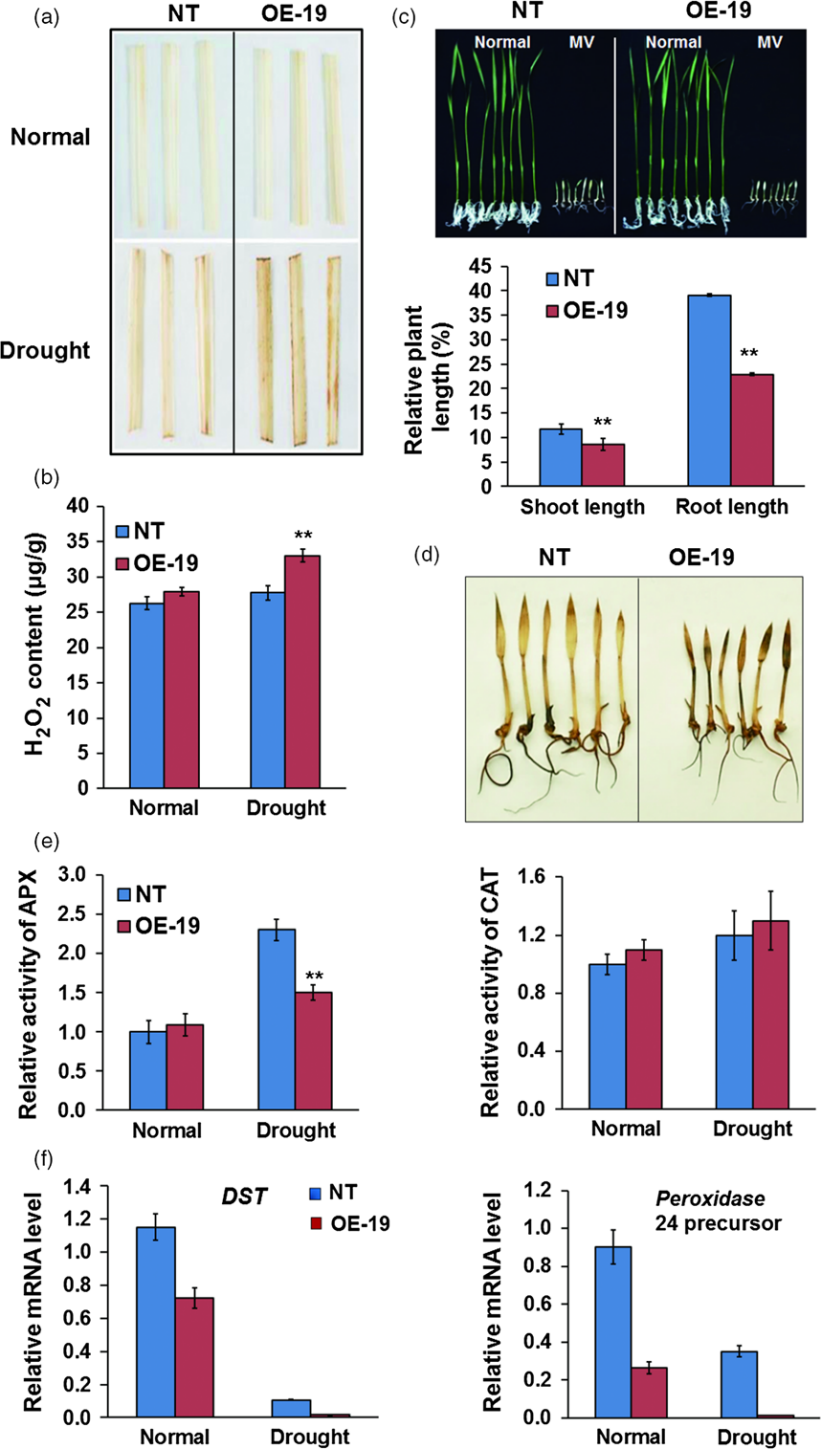
H_2_O_2_ accumulation in *OsASR5* overexpression plants. (a) 3,3φ‐Diaminobenzidine (DAB) staining for H_2_O_2_ in the leaves of *OsASR5* overexpression and NT plants under normal and drought stress conditions. (b) Quantitative measurement of H_2_O_2_ in the leaves of *OsASR5* overexpression and NT plants under normal and drought stress conditions (*n *=* *3, three plants in each repeat). (c) Growth performance and relative plant length of *OsASR5* overexpression and NT plants after MV treatment (*n *=* *3*,* five plants in each repeat). (d) DAB staining for H_2_O_2_ in the leaves of *OsASR5* overexpression and NT plants after MV treatment corresponding to C. (e) Activity of APX and CAT in the leaves of *OsASR5* overexpression and NT plants under normal and drought stress conditions (*n *=* *3). (f) Expression of *DST* and *peroxidase 24 precursor* in the leaves of *OsASR5* overexpression and NT plants under normal and drought stress conditions. Data are mean ± SE. ** indicates significant difference at *P* < 0.01 probability.

As ABA induced H_2_O_2_ generation in *Arabidopsis* (Pei *et al*., 2000; Zhang *et al*., 2001), and the accumulation of H_2_O_2_ resulting in stomatal closure was reported in rice (Huang *et al*., 2009; You *et al*., 2013), the H_2_O_2_ levels in the leaves of *OsASR5* overexpression and NT plants were necessarily to be examined. A higher production of H_2_O_2_ was detected in the leaves of *OsASR5* overexpression plants under drought stress conditions (Figure 7a,b), suggesting that the accumulation of H_2_O_2_ may increase stomatal closure in *OsASR5* overexpression plants.

Overexpression of *OsASR5* inducing H_2_O_2_ accumulation also prompted us to determine whether *OsASR5* is involved in oxidative stress response. Germinated seedlings of the *OsASR5* overexpression and NT plants were sown on 1/2 Murashige and Skoog (MS) and 1/2 MS medium containing 2 μm methyl viologen (MV). The growth rate was markedly reduced, and more H_2_O_2_ was accumulated in *OsASR5* overexpression seedlings as compared with NT seedlings after oxidative stress treatment, while no obvious changes were observed under normal conditions (Figure 7c,d). These results suggest that overexpression of *OsASR5* is sensitive to oxidative stress.

To understand the mechanism of *OsASR5* in modulating H_2_O_2_ homeostasis, the activities of H_2_O_2_‐scavenging enzymes were measured in *OsASR5* overexpression and NT plants. The results showed that the activity of *APX* was reduced in *OsASR5* overexpression plants as compared with NT plants under drought stress conditions (Figure 7e). Because *peroxidase 24 precursor* that encodes a peroxidase to scavenge H_2_O_2_ was regulated by *DST*, a negative regulator of H_2_O_2_ accumulation (Huang *et al*., 2009), expression levels of *DST* and *peroxidase 24 precursor* were analysed in *OsASR5* overexpression plants. The results revealed the expression of *DST* and *peroxidase 24 precursor* was significantly repressed in *OsASR5* overexpression plants as compared with NT plants (Figure 7f). These results demonstrate that *OsASR5* could regulate H_2_O_2_ homeostasis by affecting the activity of H_2_O_2_‐scavenging enzyme, APX, and suppressing *DST* and its downstream gene, *peroxidase 24 precursor*.

### The osasr5 mutant is sensitive to drought stress

**Figure 8:**
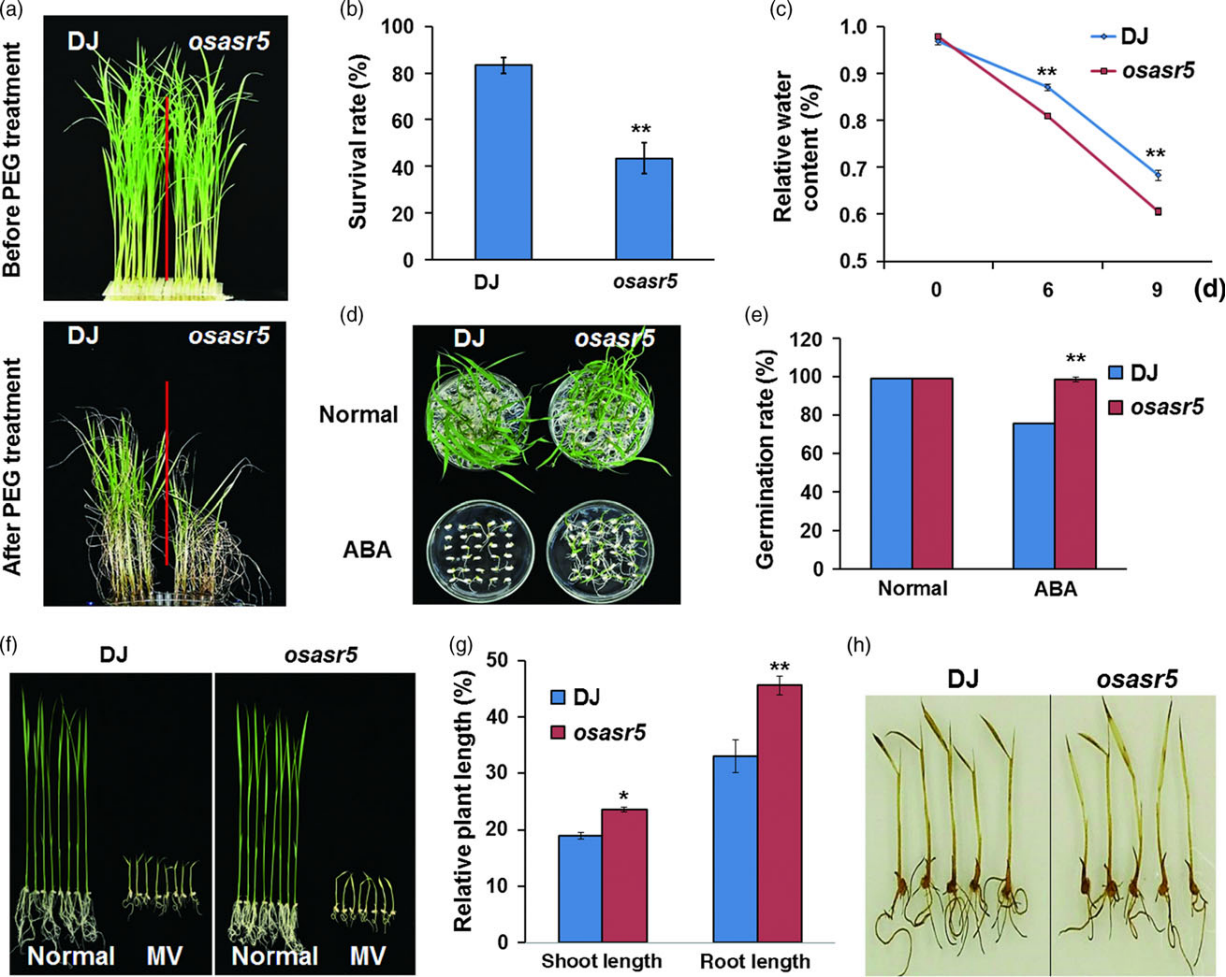
Increased drought and reduced ABA and oxidative sensitivities of the loss‐of‐function *osasr*5 mutant. (a) Physiological dehydration stress assay of *osasr*5 mutant and DJ with 15% PEG6000 treatment. (b) Survival rates of *osasr*5 mutant and DJ after dehydration stress (*n *=* *3). (c) Relative water content of *osasr*5 mutant and DJ with 15% PEG6000 treatment observed at three different time intervals (0, 3 and 9d). (d) Germination performance of *osasr*5 mutant and DJ under ABA treatment (*n *=* *3, 30 seeds in each repeat). (e) Germination rates of *osasr*5 mutant and DJ corresponding to d. (f) Growth performance of *osasr*5 mutant and DJ after MV treatment (*n *=* *3*,* five plants in each repeat). (g) Relative plant length of *osasr*5 mutant and DJ corresponding to f. (h) DAB staining for H_2_O_2_ in the leaves of *osasr*5 mutant and DJ corresponding to f. Data are mean ± SE. ** indicates significant difference at *P* < 0.01 probability.

To confirm the function of *OsASR5* in response to drought stress, T‐DNA insertion line, *osasr5*, was obtained. The T‐DNA was inserted in the promoter region, in 310 bp upstream of ATG (Figure S6A). Real‐time PCR analysis showed that almost no *OsASR5* transcript was detected in the insertion line, indicating that *osasr5* was true loss‐of‐function mutant line (Figure S6B). The growth of *osasr5* mutant (Dongjing background) is similar to that of Dongjing (DJ); however, *osasr5* mutant was hypersensitive to drought stress by 15% PEG6000 treatment (Figure 8a). The survival rate of *osasr5* mutant was only 39%–44%, while 80%–83% of the DJ was recovered (Figure 8b). Low relative water content was observed in the leaves of *osasr5* mutant under 15% PEG6000 treatment (Figure 8c). Moreover, *osasr5* mutant showed reduced ABA sensitivity as compared with DJ at germination stage (Figure 8d,e); *osasr5* mutant also showed the increased growth rate and reduced H_2_O_2_ accumulation after oxidative stress treatment (Figure 8f). Together, these results reconfirm the function of *OsASR5* in drought stress tolerance.

### *OsASR5* interacts with stress‐related proteins in chloroplast

**Figure 9:**
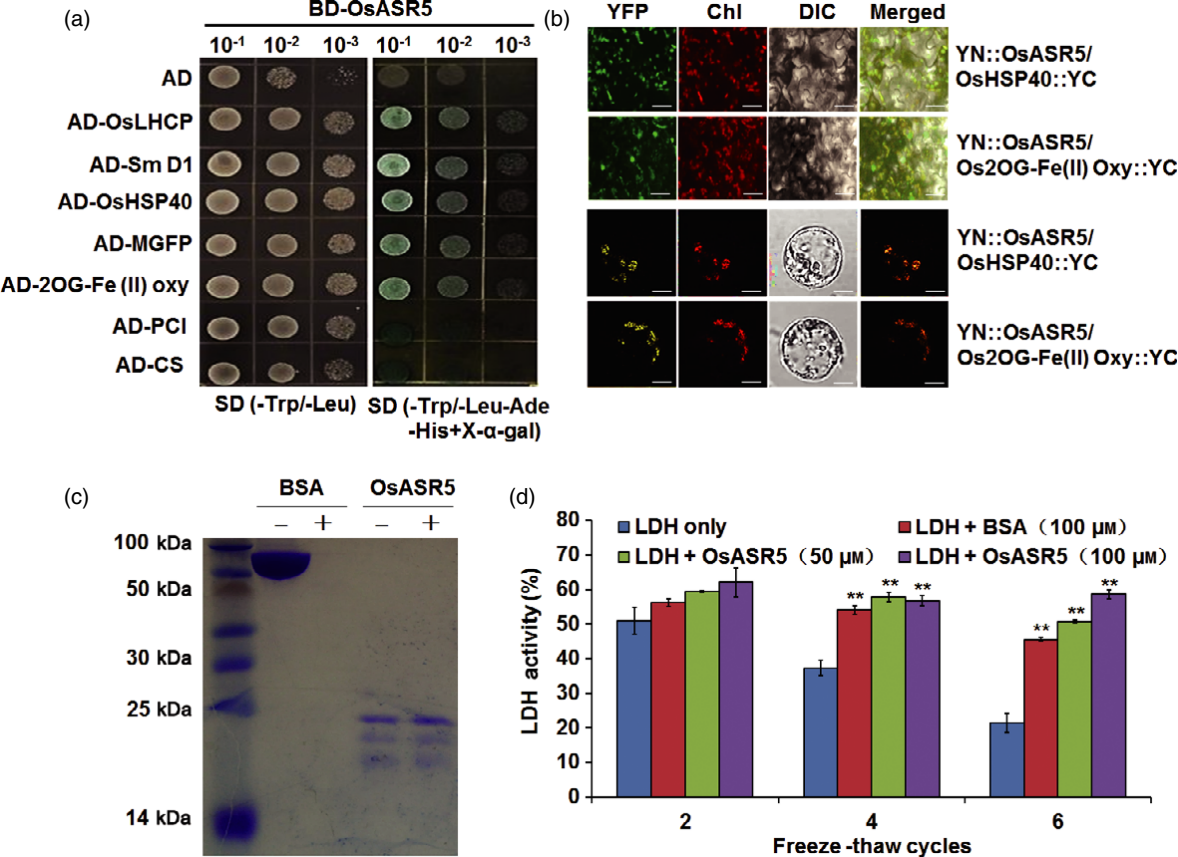
Interaction proteins and molecular chaperone activity of OsASR5. (a) Y2H assay of OsASR5 interacting proteins. BD‐OsASR5 co‐transformed with AD empty vector is used as negative control. Three different concentrations of yeast cells were grown on control plate (‐Trp/‐Leu) and selective plate (‐Trp/‐Leu/‐Ade/‐His/X‐α‐gal). (b) BiFC assay for the *in vivo* interaction of OsASR5 with OsHSP40 and with OsFe(II) Oxy in tobacco epidermal cells (upper) and rice protoplast (lower). Bar, 10 μm. (c) OsASR5 and BSA (control) were not (−) or were (+) boiled at 100 °C for 30 min (*n *=* *3). (d) LDH activity in the presence or absence of OsASR5 during cycles of freeze–thaw treatments (*n *=* *3). Data are mean ± SE. ** indicates significant difference at *P* < 0.01 probability.

In our homologous *in vivo* system with transgenic rice protoplast expressing OsASR5‐GFP fusion protein, we verified that the OsASR5 protein was localized in chloroplast and nucleus (Figure S7). As ASR proteins were reported to bind with DNA motif (Arenhart *et al*., 2014; Ricardi *et al*., 2014), it is necessary to analyse the transcription activity of OsASR5. However, the expression of BD (GAL4‐binding domain)‐OsASR5 fusion protein in yeast did not lead to reporter gene expression, and did not form homodimers to function (Figure S8), which indicated that OsASR5 has no transcriptional activity in yeast. To further elucidate the function of OsASR5, a cDNA library of IRAT109 treated with drought stress was constructed for screening OsASR5‐interacting proteins by yeast two‐hybrid (Y2H) system. Using the full‐length OsASR5 as bait, 24 positive clones were identified, and seven of them were confirmed to be unique interacting proteins (Figure 9a, Table S1). The interactions of OsASR5 with a heat‐shock protein, HSP40, and with a 2OG‐Fe (II) oxygenase family protein in the chloroplasts of tobacco epidermal cells and rice protoplast were confirmed by bimolecular fluorescence complementation (BiFC) assay (Figure 9b).

### *OsASR5* functions as chaperone‐like protein

ASR proteins are low molecular weight charged and hydrophilic proteins (Goldgur *et al*., 2007; Gonzalez and Iusem, 2014), while hydrophilic proteins were shown to possess chaperone‐like activity (Garay‐Arroyo *et al*., 2000). OsASR5 was predicted to be a hydrophilic protein (data not shown) and was heat stable (Figure 9c), which indicates that OsASR5 is not likely to aggregate during high temperature treatment or boiling. We also examined whether OsASR5 exhibits chaperone activity to protect protein from inactivation. The activity of LDH in the presence or absence of OsASR5 *in vitro* was detected during cycles of freeze–thaw treatment. The activity of LDH was significantly reduced after four cycles of freeze–thaw, while the enzyme activity was markedly retained in the presence of OsASR5 (Figure 9d). It is worth to note that the effect of enzyme protection by OsASR5 is superior to BSA, a cryoprotectant, after six cycles of freeze–thaw. Thus, these results indicate that OsASR5 can function as chaperone‐like protein and stabilize proteins against inactivation.

## Discussion

### *OsASR* genes preferentially expressed in UR are probably drought‐responsive genes in rice

Previous studies reported comparative expression profiles of UR and LR under drought stress conditions using cDNA microarray technology (Ding *et al*., 2013; Lenka *et al*., 2011; Wang *et al*., 2007). In addition, differently expressed genes in the two genotypes were identified, and several of them were currently proved to be involved in drought response. For instance, *SNAC1*,* OsLEA3‐1* and *OsMIOX* were strongly induced in UR variety by drought stress as compared with LR variety, and overexpression of these genes separately in LR variety showed significantly improved drought tolerance (Duan *et al*., 2012; Hu *et al*., 2006; Xiao *et al*., 2007). In recent years, the expression patterns of *ASR* genes both in tissue‐specific and in abiotic stresses were characterized by several groups (Joo *et al*., 2013; Philippe *et al*., 2010); however, we firstly analysed the expression changes of the rice *ASR* gene family between UR variety and LR variety. As described in Figure S1, *OsASR3* was up‐regulated in UR variety, *OsASR5* and *OsASR6* were strongly induced by drought stress in UR variety, and the expression levels of these genes were 4.2‐ to 89.6‐fold higher in UR variety than those in LR variety during drought stress. Based on our findings and the knowledge gathered, we could deduce that *OsASR3*,* OsASR5* and *OsASR6* up‐regulated in UR variety were possibly drought stress‐responsive genes in rice. In order to confirm the hypothesis, these *ASR* genes were overexpressed into the *japonica* rice, Nipponbare, separately. And the role of *OsASR5* in response to drought stress was identified firstly.

### *OsASR5* plays a positive role in drought stress response

It is widely accepted that the genes induced by abiotic stresses may play positive roles in abiotic stress tolerances. The transcription of *OsASR5* was strongly induced by dehydration, high salinity, cold, ABA and ethylene treatments. *OsASR5* overexpression lines showed improved growth performance under simulated osmotic stress conditions brought by NaCl or mannitol treatment, and enhanced the survival rate under dehydration conditions created by PEG treatment or dry soil conditions brought by restricting irrigation. Expression of *OsASR5* also enhanced osmotic and drought stress tolerances in *E. coli* and *Arabidopsis*, respectively. Furthermore, overexpression of *OsASR5* showed no obvious changes in morphological phenotype in rice and *Arabidopsis* transgenic lines under normal conditions. These results suggest that *OsASR5* is a positive regulator of the responses to drought, osmotic and dehydration stresses, implying the usefulness of *OsASR5* in genetic improvement of abiotic stress tolerance in several crop species.

### *OsASR5* confers tolerance to drought stress through a stomatal closure pathway associated with ABA and H2O2 signalling

Stomata control uptake of CO_2_ for photosynthesis and restrict water loss by modulating transpiration, thereby playing crucial roles in abiotic stress tolerance (Hetherington and Woodward, 2003; Schroeder *et al*., 2001). Due to the essential roles of the stomata for plants, the molecular mechanisms of stomatal movement integrated by phytohormone, environmental signalling and many ion channels have been frequently studied in *Arabidopsis* (Daszkowska‐Golec and Szarejko, 2013). So far, a total of seven drought‐responsive genes that regulating stomatal movement have been identified in rice (Gao *et al*., 2011; Hu *et al*., 2006; Huang *et al*., 2009; Manavalan *et al*., 2012; Wei *et al*., 2014; You *et al*., 2013; Zhang *et al*., 2011). Among which, *SNAC1*,* OsSDIR1*,* hrf1*,* SQS* and *OsCPK9* were characterized to be sensitive to ABA, modulating stomatal movement possibly through an ABA‐dependent pathway (Gao *et al*., 2011; Hu *et al*., 2006; Manavalan *et al*., 2012; Wei *et al*., 2014; Zhang *et al*., 2011a), while *DST* and *OsSRO1* regulated stomatal movement due to the accumulation of H_2_O_2_ through an ABA‐independent pathway (Huang *et al*., 2009; You *et al*., 2013). Therefore, knowledge on control of stomatal closure and opening remains fragmented in rice. In this study, *OsASR5* was strongly induced by exogenous ABA treatment, and the endogenous ABA level of *OsASR5* overexpression plants under drought stress conditions was much higher than that of NT. Furthermore, *OsASR5* overexpression plants were more sensitive, and *osasr5* mutant was more insensitive to exogenous ABA treatment than that of their WT, respectively. These results indicated that the *OsASR5* was involved in an ABA‐dependent pathway.

H_2_O_2_ generation was dependent on ABA concentration and was essential for ABA‐induced stomatal closure in plants (Kwak *et al*., 2003; Wang and Song, 2008; Zhang *et al*., 2001). We found a higher accumulation of H_2_O_2_ along with the increased ABA level, and the coincidence of reduced rate of water loss with increased stomatal closure in *OsASR5* overexpression plants under drought stress conditions. To our knowledge, we suggest that *OsASR5* modulates stomatal closure probably due to the H_2_O_2_ accumulation through ABA‐dependent pathway under drought stress conditions.

### Possible functions of the OsASR5 protein in chloroplast

ASR proteins were previously reported to be localized in both the cytosol and nucleus (Chen *et al*., 2011; Ricardi *et al*., 2012; Takasaki *et al*., 2008), only in the nucleus (Hu *et al*., 2013; Hwan *et al*., 2012) or in multiple cellular compartments such as nucleus, cytoplasm and chloroplasts (Arenhart *et al*., 2014). However, the precise function for the localization of ASR proteins in these subcellular compartments is not clear. We identified HSP40 and a 2OG‐Fe (II) oxygenase family protein that interacted with OsASR5 in the chloroplasts of tobacco epidermal cells and rice protoplasts, separately. This is the first report on the interaction of ASR proteins in the chloroplast. HSPs are stimulated in response to abiotic stress and play an important role in protecting plants against many stresses (Alvim *et al*., 2001; Cho and Hong, 2006; Sato and Yokoya, 2008; Timperio *et al*., 2008; Wang *et al*., 2014). A 2OG‐Fe (II) oxygenase family protein in rice affects water transport in leaves by affecting the composition and structure of leaf secondary cell walls (Fang *et al*., 2012). These evidences imply that OsASR5‐interacting proteins, HSP40 and 2OG‐Fe (II) oxygenase family protein may be playing important roles in response to water stress.

Abiotic stress may result in protein aggregation and degradation. Plants use a number of mechanisms to protect protein from inactivation, including chaperones and chaperone‐like proteins, and low molecular weight organic molecules (Konrad and Bar‐Zvi, 2008). *In vitro* assay with purified OsASR5 protein, we confirmed that OsASR5 could protect LDH from cold‐induced inactivation, function as chaperone‐like protein. Therefore, we could conclude that OsASR5 may function as molecular chaperone for the HSP40 and 2OG‐Fe (II) oxygenase family protein in chloroplast, and possibly prevent HSP40 and 2OG‐Fe (II) oxygenase family protein from inactivation under drought stress conditions.

**Figure 10:**
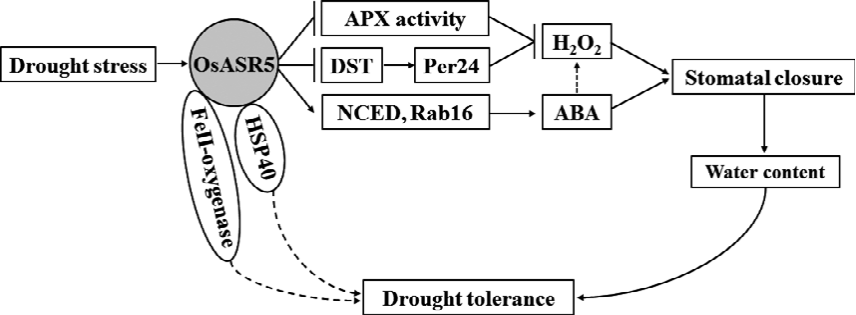
A proposed model explaining the function of OsASR5 in the regulation of stomatal status and drought stress tolerance. Under drought stress, the expression of OsASR5 was up‐regulated,resulting in up‐regulation of ABA biosynthesis and responsive genes,such as *OsNCED4* and *OsNCED5*, *RAB16A* and *RAB16C*, leading to ABA accumulation and increased sensitivity to exogenous ABA. Up‐regulation of OsASR5 also affected the activity of H_2_O_2_‐scavenging enzyme, APX, and suppressed *DST* and its downstream gene, *peroxidase 24 precursor*, leading to H_2_O_2_ accumulation. ABA and H_2_O_2_ accumulation promotes stomatal closure, resulting in increased water content and finally enhancing drought tolerance. Furthermore, OsASR5 functioned as molecular chaperone and interacted with HSP40 and 2OG‐Fe (II) oxygenase family protein, may prevent those drought stress‐related proteins from inactivation under drought stress conditions. However, the function of those interacted proteins for drought stress tolerance remains to be elucidated in future studies.

Based on our knowledge, we try to summarize a model to explain the role of OsASR5 in improving drought stress tolerance in plant (Figure 10). In conclusion, *OsASR5* has multiple roles in the regulation of drought stress tolerance by increasing ABA and H_2_O_2_ accumulation, thus leading to stomatal closure and reduce water loss, besides by acting as chaperone‐like protein that possibly protects some drought stress‐related proteins from inactivation under drought stress conditions. Furthermore, overexpression of *OsASR5* did not alter the morphological phenotype of the transgenic lines. Therefore, through this study, we could successfully identify a gene, *OsASR5*, which may be potentially useful for engineering drought tolerance in plant.

## Experimental procedures

### Plant materials and growth conditions

The UR variety, IRAT109, and LR variety, Nipponbare, were used in this study. The *japonica* rice variety, DJ and mutant *osasr5* seeds were obtained from the POSTECH RISD (http://www.postech.ac.kr/life/pfg/risd/). Seeds of IRAT109 and Nipponbare were germinated at 32 °C for 2 days and then grown in Hoagland nutrient solution under controlled conditions with 28 ± 2 °C temperature, 200 μmol/m^2^s^2^ light intensity with 14‐h light/10‐h dark photoperiod and 80% relative humidity. Four‐week‐old seedlings were subjected to different treatments with 15% PEG6000 (w/v), 200 mm NaCl, low temperature (4 °C), 100 μm ABA, 2 mm ethylene and drought by stopping irrigation. The leaf tissues were harvested at 0‐, 0.5‐, 1‐, 2‐, 3‐, 6‐, 10‐, 16‐ and 24‐h time points for PEG, low temperature, ABA and ethylene treatments. Likewise, leaf tissues were harvested at 0‐, 0.5‐, 1‐, 2‐, 3‐, 6‐, 10‐ and 16‐h time points for NaCl treatment and at 0‐, 4‐, 8‐, 11‐, 14‐, 17‐, 21‐, 25‐ and 28‐d time points for drought treatment. All these harvested leaf samples were then rapidly freezed in liquid nitrogen and stored at −80 °C for further expression analysis of *OsASR5* between UR and LR.

### Osmotic, drought and oxidative stress treatments

For osmotic stress treatment, the seeds of T_3_ transgenic and NT lines were germinated on 1/2 MS medium under 14‐h light (28 °C)/10‐h dark (25 °C) photoperiod conditions for 5 days and transplanted to 1/2 MS medium containing 150 mm NaCl and 250 mm mannitol, respectively. The shoot length, root length and fresh weight of transgenic lines and NT plants were measured after 7 days. For dehydration treatment, 3‐week‐old seedlings of *OsASR5* overexpression and NT plants, mutant *osasr5* and DJ plants grown in normal Hoagland solution were treated with 15% PEG6000 solution for 14 days and then recovered in normal Hoagland solution for 7 days. The survival rates of each line were examined. For drought treatment, 2‐week‐old seedlings of *OsASR5* overexpression *Arabidopsis* transgenic lines and WT grown at 10‐h light (22 °C)/14‐h dark (18 °C) photoperiod in flowerpots with soil and vermiculite (1 : 2) were not irrigated. After 3 weeks of stopping irrigation, the seedlings were observed for recovery by rewatering for 4 days. Fresh leaf numbers of *OsASR5* overexpression *Arabidopsis* transgenic lines and WT before and after drought stress were examined. One‐month‐old seedlings of the *OsASR5* overexpression rice transgenic lines and NT plants grown in flowerpots with soil and vermiculite (1 : 1) were not irrigated. After 1 week of stopping irrigation, the seedlings were observed for recovery by rewatering for 2 weeks. Seedlings were regarded as survivals if the fresh and green young leaves emerged after water supply. The survival ratio was calculated according to the number of survival plants over the treated plants in each flowerpot. For oxidative treatment, the seeds of *OsASR5* overexpression and NT plants germinated on normal 1/2 MS medium were transplanted to 1/2 MS medium containing 2 μm MV, and the plant length was measured at 5 days after transplanting.

### Imaging of stomatal opening and measurement of stomatal conductance

Leaves of 1‐month‐old *OsASR5* overexpression and NT plants with drought treatment (without irrigation for 3 days) or normal growth were detached and directly fixed by 2.5% glutaraldehyde. The stomatal pictures were obtained using a scanning electron microscopy (JSM‐6390lv, JEOL, Japan), and the percentages of stomatal completely open, partially open and completely close were calculated. The second fully expanded leaves, counting from the top of the same plants used for imaging stomata, were applied to measure stomatal conductance with a portable gas analysis system (LI‐COR 6400, LI‐COR, Inc.).

### Endogenous ABA level and exogenous ABA sensitivity assay

Endogenous ABA levels were determined according to the method as described previously (Xiong *et al*., 2014). To test the ABA sensitivity at germination stage, seeds of *OsASR5* overexpression and NT, *osasr5* and DJ lines were germinated on 1/2 MS medium containing 2.5 μm ABA and the germination rates were calculated at the fifth day after initiation. To test the sensitivity at postgermination stage, the seeds of overexpression and NT plants germinated on normal 1/2 MS medium were transplanted to 1/2 MS medium containing 2.5 μm ABA. The shoot length and root length of each seedlings were measured after 7 days of the ABA treatment at 14‐h light (28 °C)/10‐h dark (25 °C) photoperiod.

## Other methods

Details of the methods for RNA isolation and qRT‐PCR analysis, plasmid construction and plant transformation, subcellular localization, physiological and biochemical indexes assay, transactivation, yeast two‐hybrid and BiFC assays are available in supplementary methods at *PBJ* online.

## Supporting information


**Figure S1** Drought inducible expression of *ASR* genes in UR and LR varieties.
**Figure S2** The temporal and spatial expression pattern of *OsASR5* in the transgenic lines harbouring a fusion gene of *Pro*_*OsASR5*_:*OsASR5‐*GFP.
**Figure S3** RT‐PCR analysis of *OsASR5* transcript levels in different *Arabidopsis* transgenic lines.
**Figure S4** Transcription levels of *OsASR5* in *OsASR5* overexpression rice transgenic lines.
**Figure S5** Free proline and soluble sugar contents of *OsASR5* overexpression and NT plants under 15% PEG6000 treatment.
**Figure S6** Identification of *osasr5* T‐DNA insertion mutant.
**Figure S7** Subcellular localization of OsASR5‐GFP fusion protein.
**Figure S8** OsASR5 transcriptional activation and homodimerization analysis.

**Table S1** OsASR5 interacting proteins identified in yeast two‐hybrid screening.

**Appendix S1** Supplementary methods.

## References

[pbi12601-bib-0001] Alvim, F.C. , Carolino, S.M. , Cascardo, J.C. , Nunes, C.C. , Martinez, C.A. , Otoni, W.C. and Fontes, E.P. (2001) Enhanced accumulation of BiP in transgenic plants confers tolerance to water stress. Plant Physiol. 126, 1042–1054. 11457955 10.1104/pp.126.3.1042 PMC116461

[pbi12601-bib-0002] Arenhart, R.A. , Bai, Y. , de Oliveira, L.F. , Neto, L.B. , Schunemann, M. , Maraschin Fdos, S. , Mariath, J. *et al*. (2014) New insights into aluminum tolerance in rice: the ASR5 protein binds the STAR1 promoter and other aluminum‐responsive genes. Mol. Plant, 7, 709–721. 24253199 10.1093/mp/sst160 PMC3973494

[pbi12601-bib-0003] Cakir, B. , Agasse, A. , Gaillard, C. , Saumonneau, A. , Delrot, S. and Atanassova, R. (2003) A grape ASR protein involved in sugar and abscisic acid signaling. Plant Cell, 15, 2165–2180. 12953118 10.1105/tpc.013854 PMC181338

[pbi12601-bib-0004] Chen, J.Y. , Liu, D.J. , Jiang, Y.M. , Zhao, M.L. , Shan, W. , Kuang, J.F. and Lu, W.J. (2011) Molecular characterization of a strawberry FaASR gene in relation to fruit ripening. PLoS ONE, 6, e24649. 21915355 10.1371/journal.pone.0024649 PMC3167850

[pbi12601-bib-0005] Cho, E.K. and Hong, C.B. (2006) Over‐expression of tobacco NtHSP70‐1 contributes to drought‐stress tolerance in plants. Plant Cell Rep. 25, 349–358. 16365678 10.1007/s00299-005-0093-2

[pbi12601-bib-0006] Dai, J.R. , Liu, B. , Feng, D.R. , Liu, H.Y. , He, Y.M. , Qi, K.B. , Wang, H.B. *et al*. (2011) MpAsr encodes an intrinsically unstructured protein and enhances osmotic tolerance in transgenic Arabidopsis. Plant Cell Rep. 30, 1219–1230. 21327389 10.1007/s00299-011-1030-1

[pbi12601-bib-0007] Daszkowska‐Golec, A. and Szarejko, I. (2013) Open or close the gate – stomata action under the control of phytohormones in drought stress conditions. Front. Plant Sci. 4, 138. 23717320 10.3389/fpls.2013.00138 PMC3652521

[pbi12601-bib-0008] Ding, X. , Li, X. and Xiong, L. (2013) Insight into differential responses of upland and paddy rice to drought stress by comparative expression profiling analysis. Int. J. Mol. Sci. 14, 5214–5238. 23459234 10.3390/ijms14035214 PMC3634487

[pbi12601-bib-0009] Duan, J. , Zhang, M. , Zhang, H. , Xiong, H. , Liu, P. , Ali, J. , Li, J. *et al*. (2012) OsMIOX, a myo‐inositol oxygenase gene, improves drought tolerance through scavenging of reactive oxygen species in rice (*Oryza sativa* L.). Plant Sci. 196, 143–151. 23017909 10.1016/j.plantsci.2012.08.003

[pbi12601-bib-0010] Fang, L. , Zhao, F. , Cong, Y. , Sang, X. , Du, Q. , Wang, D. , Li, Y. *et al*. (2012) Rolling‐leaf14 is a 2OG‐Fe (II) oxygenase family protein that modulates rice leaf rolling by affecting secondary cell wall formation in leaves. Plant Biotechnol. J. 10, 524–532. 22329407 10.1111/j.1467-7652.2012.00679.x

[pbi12601-bib-0011] Gao, T. , Wu, Y. , Zhang, Y. , Liu, L. , Ning, Y. , Wang, D. , Tong, H. *et al*. (2011) OsSDIR1 overexpression greatly improves drought tolerance in transgenic rice. Plant Mol. Biol. 76, 145–156. 21499841 10.1007/s11103-011-9775-z

[pbi12601-bib-0012] Garay‐Arroyo, A. , Colmenero‐Flores, J.M. , Garciarrubio, A. and Covarrubias, A.A. (2000) Highly hydrophilic proteins in prokaryotes and eukaryotes are common during conditions of water deficit. J. Biol. Chem. 275, 5668–5674. 10681550 10.1074/jbc.275.8.5668

[pbi12601-bib-0013] Goldgur, Y. , Rom, S. , Ghirlando, R. , Shkolnik, D. , Shadrin, N. , Konrad, Z. and Bar‐Zvi, D. (2007) Desiccation and zinc binding induce transition of tomato abscisic acid stress ripening 1, a water stress‐ and salt stress‐regulated plant‐specific protein, from unfolded to folded state. Plant Physiol. 143, 617–628. 17189335 10.1104/pp.106.092965 PMC1803749

[pbi12601-bib-0014] Gonzalez, R.M. and Iusem, N.D. (2014) Twenty years of research on Asr (ABA‐stress‐ripening) genes and proteins. Planta, 239, 941–949. 24531839 10.1007/s00425-014-2039-9

[pbi12601-bib-0015] Henry, I.M. , Carpentier, S.C. , Pampurova, S. , Van Hoylandt, A. , Panis, B. , Swennen, R. and Remy, S. (2011) Structure and regulation of the Asr gene family in banana. Planta 234, 785–798. 21630042 10.1007/s00425-011-1421-0 PMC3180632

[pbi12601-bib-0016] Hetherington, A.M. and Woodward, F.I. (2003) The role of stomata in sensing and driving environmental change. Nature, 424, 901–908. 12931178 10.1038/nature01843

[pbi12601-bib-0017] Hsu, Y.F. , Yu, S.C. , Yang, C.Y. and Wang, C.S. (2011) Lily ASR protein‐conferred cold and freezing resistance in Arabidopsis. Plant Physiol. Biochem. 49, 937–945. 21803593 10.1016/j.plaphy.2011.07.002

[pbi12601-bib-0018] Hu, H. and Xiong, L. (2014) Genetic engineering and breeding of drought‐resistant crops. Annu. Rev. Plant Biol. 65, 715–741. 24313844 10.1146/annurev-arplant-050213-040000

[pbi12601-bib-0019] Hu, H. , Dai, M. , Yao, J. , Xiao, B. , Li, X. , Zhang, Q. and Xiong, L. (2006) Overexpressing a NAM, ATAF, and CUC (NAC) transcription factor enhances drought resistance and salt tolerance in rice. Proc. Natl Acad. Sci. USA, 103, 12987–12992. 16924117 10.1073/pnas.0604882103 PMC1559740

[pbi12601-bib-0020] Hu, W. , Huang, C. , Deng, X. , Zhou, S. , Chen, L. , Li, Y. , Wang, C. *et al*. (2013) TaASR1, a transcription factor gene in wheat, confers drought stress tolerance in transgenic tobacco. Plant Cell Environ. 36, 1449–1464. 23356734 10.1111/pce.12074

[pbi12601-bib-0021] Huang, J.C. , Lin, S.M. and Wang, C.S. (2000) A pollen‐specific and desiccation‐associated transcript in *Lilium longiflorum* during development and stress. Plant Cell Physiol. 41, 477–485. 10845461 10.1093/pcp/41.4.477

[pbi12601-bib-0022] Huang, X.Y. , Chao, D.Y. , Gao, J.P. , Zhu, M.Z. , Shi, M. and Lin, H.X. (2009) A previously unknown zinc finger protein, DST, regulates drought and salt tolerance in rice via stomatal aperture control. Genes Dev. 23, 1805–1817. 19651988 10.1101/gad.1812409 PMC2720257

[pbi12601-bib-0023] Hwan, Y.S. , Hyon, K.I.M.S. , Berberich, T. and Kusano, T. (2012) Identification and properties of a small protein that interacts with a tobacco bZIP‐type transcription factor TBZF. Plant Biotechnol. 29, 395–399.

[pbi12601-bib-0024] Jha, B. , Lal, S. , Tiwari, V. , Yadav, S.K. and Agarwal, P.K. (2012) The SbASR‐1 gene cloned from an extreme halophyte Salicornia brachiata enhances salt tolerance in transgenic tobacco. Mar. Biotechnol. (NY) 14, 782–792. 22639284 10.1007/s10126-012-9442-7

[pbi12601-bib-0025] Joo, J. , Lee, Y.H. , Kim, Y.K. , Nahm, B.H. and Song, S.I. (2013) Abiotic stress responsive rice ASR1 and ASR3 exhibit different tissue‐dependent sugar and hormone‐sensitivities. Mol. Cells, 35, 421–435. 23620302 10.1007/s10059-013-0036-7 PMC3887869

[pbi12601-bib-0026] Kalifa, Y. , Perlson, E. , Gilad, A. , Konrad, Z. , Scolnik, P.A. and Bar‐Zvi, D. (2004) Over‐expression of the water and salt stress‐regulated Asr1 gene confers an increased salt tolerance. Plant Cell Environ. 27, 1459–1468.

[pbi12601-bib-0027] Kim, S.J. , Lee, S.C. , Hong, S.K. , An, K. , An, G. and Kim, S.R. (2009) Ectopic expression of a cold‐responsive OsAsr1 cDNA gives enhanced cold tolerance in transgenic rice plants. Mol. Cells 27, 449–458. 19390826 10.1007/s10059-009-0055-6

[pbi12601-bib-0028] Konrad, Z. and Bar‐Zvi, D. (2008) Synergism between the chaperone‐like activity of the stress regulated ASR1 protein and the osmolyte glycine‐betaine. Planta 227, 1213–1219. 18270732 10.1007/s00425-008-0693-5

[pbi12601-bib-0029] Kwak, J.M. , Mori, I.C. , Pei, Z.M. , Leonhardt, N. , Torres, M.A. , Dangl, J.L. , Bloom, R.E. *et al*. (2003) NADPH oxidase AtrbohD and AtrbohF genes function in ROS‐dependent ABA signaling in Arabidopsis. EMBO J. 22, 2623–2633. 12773379 10.1093/emboj/cdg277 PMC156772

[pbi12601-bib-0030] Lee, S.C. and Luan, S. (2012) ABA signal transduction at the crossroad of biotic and abiotic stress responses. Plant Cell Environ. 35, 53–60. 21923759 10.1111/j.1365-3040.2011.02426.x

[pbi12601-bib-0031] Lenka, S.K. , Katiyar, A. , Chinnusamy, V. and Bansal, K.C. (2011) Comparative analysis of drought‐responsive transcriptome in Indica rice genotypes with contrasting drought tolerance. Plant Biotechnol. J. 9, 315–327. 20809928 10.1111/j.1467-7652.2010.00560.x

[pbi12601-bib-0032] Luo, L.J. (2010) Breeding for water‐saving and drought‐resistance rice (WDR) in China. J. Exp. Bot. 61, 3509–3517. 20603281 10.1093/jxb/erq185

[pbi12601-bib-0033] Manavalan, L.P. , Chen, X. , Clarke, J. , Salmeron, J. and Nguyen, H.T. (2012) RNAi‐mediated disruption of squalene synthase improves drought tolerance and yield in rice. J. Exp. Bot. 63, 163–175. 21926092 10.1093/jxb/err258 PMC3245457

[pbi12601-bib-0034] Maskin, L. , Gudesblat, G.E. , Moreno, J.E. , Carrari, F.O. , Frankel, N. , Sambade, A. , Rossi, M. *et al*. (2001) Differential expression of the members of the Asr gene family in tomato (*Lycopersicon esculentum*). Plant Sci. 161, 739–746.

[pbi12601-bib-0035] Mustilli, A.C. , Merlot, S. , Vavasseur, A. , Fenzi, F. and Giraudat, J. (2002) Arabidopsis OST1 protein kinase mediates the regulation of stomatal aperture by abscisic acid and acts upstream of reactive oxygen species production. Plant Cell, 14, 3089–3099. 12468729 10.1105/tpc.007906 PMC151204

[pbi12601-bib-0036] Nguyen, H.T. , Babu, R.C. and Blum, A. (1997) Breeding for drought resistance in rice: physiology and molecular genetics considerations. Crop Sci. 37, 1426–1434.

[pbi12601-bib-0037] Pei, Z.M. , Murata, Y. , Benning, G. , Thomine, S. , Klusener, B. , Allen, G.J. , Grill, E. *et al*. (2000) Calcium channels activated by hydrogen peroxide mediate abscisic acid signalling in guard cells. Nature, 406, 731–734. 10963598 10.1038/35021067

[pbi12601-bib-0038] Perez‐Diaz, J. , Wu, T.M. , Perez‐Diaz, R. , Ruiz‐Lara, S. , Hong, C.Y. and Casaretto, J.A. (2014) Organ‐ and stress‐specific expression of the ASR genes in rice. Plant Cell Rep. 33, 61–73. 24085307 10.1007/s00299-013-1512-4

[pbi12601-bib-0039] Philippe, R. , Courtois, B. , McNally, K.L. , Mournet, P. , El‐Malki, R. , Le Paslier, M.C. , Fabre, D. *et al*. (2010) Structure, allelic diversity and selection of Asr genes, candidate for drought tolerance, in *Oryza sativa* L. and wild relatives. TAG 121, 769–787. 20454772 10.1007/s00122-010-1348-z

[pbi12601-bib-0040] Price, A.H. , Cairns, J.E. , Horton, P. , Jones, H.G. and Griffiths, H. (2002) Linking drought‐resistance mechanisms to drought avoidance in upland rice using a QTL approach: progress and new opportunities to integrate stomatal and mesophyll responses. J. Exp. Bot. 53, 989–1004. 11971911 10.1093/jexbot/53.371.989

[pbi12601-bib-0041] Ricardi, M.M. , Guaimas, F.F. , Gonzalez, R.M. , Burrieza, H.P. , Lopez‐Fernandez, M.P. , Jares‐Erijman, E.A. , Estevez, J.M. *et al*. (2012) Nuclear import and dimerization of tomato ASR1, a water stress‐inducible protein exclusive to plants. PLoS ONE, 7, e41008. 22899993 10.1371/journal.pone.0041008 PMC3416805

[pbi12601-bib-0042] Ricardi, M.M. , Gonzalez, R.M. , Zhong, S. , Dominguez, P.G. , Duffy, T. , Turjanski, P.G. , Salgado Salter, J.D. *et al*. (2014) Genome‐wide data (ChIP‐seq) enabled identification of cell wall‐related and aquaporin genes as targets of tomato ASR1, a drought stress‐responsive transcription factor. BMC Plant Biol. 14, 29. 24423251 10.1186/1471-2229-14-29 PMC3923394

[pbi12601-bib-0043] Sato, Y. and Yokoya, S. (2008) Enhanced tolerance to drought stress in transgenic rice plants overexpressing a small heat‐shock protein, sHSP17.7. Plant Cell Rep. 27, 329–334. 17968552 10.1007/s00299-007-0470-0

[pbi12601-bib-0044] Saumonneau, A. , Agasse, A. , Bidoyen, M.T. , Lallemand, M. , Cantereau, A. , Medici, A. , Laloi, M. *et al*. (2008) Interaction of grape ASR proteins with a DREB transcription factor in the nucleus. FEBS Lett. 582, 3281–3287. 18804467 10.1016/j.febslet.2008.09.015

[pbi12601-bib-0045] Saumonneau, A. , Laloi, M. , Lallemand, M. , Rabot, A. and Atanassova, R. (2012) Dissection of the transcriptional regulation of grape ASR and response to glucose and abscisic acid. J. Exp. Bot. 63, 1495–1510. 22140241 10.1093/jxb/err391

[pbi12601-bib-0046] Schroeder, J.I. , Allen, G.J. , Hugouvieux, V. , Kwak, J.M. and Waner, D. (2001) Guard cell signal transduction. Annu. Rev. Plant Physiol. Plant Mol. Biol. 52, 627–658. 11337411 10.1146/annurev.arplant.52.1.627

[pbi12601-bib-0047] Takasaki, H. , Mahmood, T. , Matsuoka, M. , Matsumoto, H. and Komatsu, S. (2008) Identification and characterization of a gibberellin‐regulated protein, which is ASR5, in the basal region of rice leaf sheaths. Mol. Genet. Genomics, 279, 359–370. 18210155 10.1007/s00438-007-0317-y

[pbi12601-bib-0048] Timperio, A.M. , Egidi, M.G. and Zolla, L. (2008) Proteomics applied on plant abiotic stresses: role of heat shock proteins (HSP). J. Proteomics. 71, 391–411. 18718564 10.1016/j.jprot.2008.07.005

[pbi12601-bib-0049] Umezawa, T. , Fujita, M. , Fujita, Y. , Yamaguchi‐Shinozaki, K. and Shinozaki, K. (2006) Engineering drought tolerance in plants: discovering and tailoring genes to unlock the future. Curr. Opin. Biotechnol. 17, 113–122. 16495045 10.1016/j.copbio.2006.02.002

[pbi12601-bib-0050] Valliyodan, B. and Nguyen, H.T. (2006) Understanding regulatory networks and engineering for enhanced drought tolerance in plants. Curr. Opin. Plant Biol. 9, 189–195. 16483835 10.1016/j.pbi.2006.01.019

[pbi12601-bib-0051] Virlouvet, L. , Jacquemot, M.P. , Gerentes, D. , Corti, H. , Bouton, S. , Gilard, F. , Valot, B. *et al*. (2011) The ZmASR1 protein influences branched‐chain amino acid biosynthesis and maintains kernel yield in maize under water‐limited conditions. Plant Physiol. 157, 917–936. 21852416 10.1104/pp.111.176818 PMC3192578

[pbi12601-bib-0052] Wang, P. and Song, C.P. (2008) Guard‐cell signalling for hydrogen peroxide and abscisic acid. New Phytol. 178, 703–718. 18373649 10.1111/j.1469-8137.2008.02431.x

[pbi12601-bib-0053] Wang, H. , Zhang, H. , Gao, F. , Li, J. and Li, Z. (2007) Comparison of gene expression between upland and lowland rice cultivars under water stress using cDNA microarray. TAG 115, 1109–1126. 17846741 10.1007/s00122-007-0637-7

[pbi12601-bib-0054] Wang, Y. , Lin, S. , Song, Q. , Li, K. , Tao, H. , Huang, J. , Chen, X. *et al*. (2014) Genome‐wide identification of heat shock proteins (Hsps) and Hsp interactors in rice: Hsp70s as a case study. BMC Genom. 15, 344. 10.1186/1471-2164-15-344 PMC4035072 24884676

[pbi12601-bib-0055] Wei, S. , Hu, W. , Deng, X. , Zhang, Y. , Liu, X. , Zhao, X. , Luo, Q. *et al*. (2014) A rice calcium‐dependent protein kinase OsCPK9 positively regulates drought stress tolerance and spikelet fertility. BMC Plant Biol. 14, 133. 24884869 10.1186/1471-2229-14-133 PMC4036088

[pbi12601-bib-0056] Xiao, B. , Huang, Y. , Tang, N. and Xiong, L. (2007) Over‐expression of a *LEA* gene in rice improves drought resistance under the field conditions. TAG 115, 35–46. 17426956 10.1007/s00122-007-0538-9

[pbi12601-bib-0057] Xiong, H. , Li, J. , Liu, P. , Duan, J. , Zhao, Y. , Guo, X. , Li, Y. *et al*. (2014) Overexpression of OsMYB48‐1, a novel MYB‐related transcription factor, enhances drought and salinity tolerance in rice. PLoS ONE, 9, e92913. 24667379 10.1371/journal.pone.0092913 PMC3965499

[pbi12601-bib-0058] Ye, N. , Jia, L. and Zhang, J. (2012) ABA signal in rice under stress conditions. Rice (NY) 5, 1. 10.1186/1939-8433-5-1 PMC3834477 24764501

[pbi12601-bib-0059] You, J. , Zong, W. , Li, X. , Ning, J. , Hu, H. , Li, X. , Xiao, J. *et al*. (2013) The SNAC1‐targeted gene OsSRO1c modulates stomatal closure and oxidative stress tolerance by regulating hydrogen peroxide in rice. J. Exp. Bot. 64, 569–583. 23202132 10.1093/jxb/ers349 PMC3542048

[pbi12601-bib-0060] Zhang, X. , Zhang, L. , Dong, F. , Gao, J. , Galbraith, D.W. and Song, C.P. (2001) Hydrogen peroxide is involved in abscisic acid‐induced stomatal closure in *Vicia faba* . Plant Physiol. 126, 1438–1448. 11500543 10.1104/pp.126.4.1438 PMC117144

[pbi12601-bib-0061] Zhang, L. , Xiao, S. , Li, W. , Feng, W. , Li, J. , Wu, Z. , Gao, X. *et al*. (2011) Overexpression of a Harpin‐encoding gene hrf1 in rice enhances drought tolerance. J. Exp. Bot. 62, 4229–4238. 21527628 10.1093/jxb/err131 PMC3153678

