# Supplementary material for: OsASR5 enhances drought tolerance through a stomatal closure pathway associated with ABA and H2O2 signalling in rice
Source: Plant Biotechnol J. 2016 Nov 11;15(2):183–96. doi: 10.1111/pbi.12601 (PMC5258865; doi:10.1111/pbi.12601)
Supplement: Supplementary file 1 — Figure S1 Drought inducible expression of ASR genes in UR and LR varieties. Figure S2 The temporal and spatial expression pattern of OsASR5 in the transgenic lines harbouring a fusion gene of Pro OsASR5 :OsASR5‐GFP. Figure S3 RT‐PCR analysis of OsASR5 transcript levels in different Arabidopsis transgenic lines. Figure S4 Transcription levels of OsASR5 in OsASR5 overexpression rice transgenic lines. Figure S5 Free proline and soluble sugar contents of OsASR5 overexpression and NT plants under 15% PEG6000 treatment. Figure S6 Identification of osasr5 T‐DNA insertion mutant. Figure S7 Subcellular localization of OsASR5‐GFP fusion protein. Figure S8 OsASR5 transcriptional activation and homodimerization analysis. [file PBI-15-183-s003.docx]

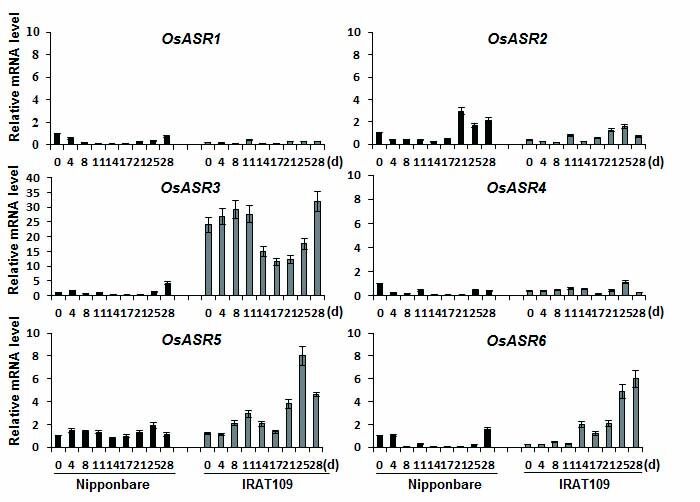


**Fig.S1** Drought inducible expression of *ASR* genes in UR and LR varieties. Four-week-old seedlings of LR variety, Nipponbare and UR variety, IRAT109 were treated with drought stress by stop irrigation. Relative expression levels of *ASR* genes were examined by real-time RCR. Error bars indicate standard error (SE) based on three replicates.


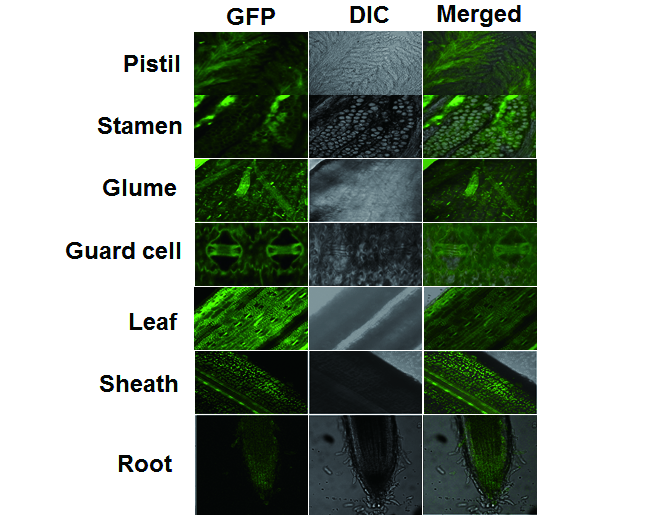


**Fig.S2** The temporal and spatial expression pattern of *OsASR5* in the transgenic lines harboring a fusion gene of *Pro_OsASR5_*:*OsASR5-*GFP.


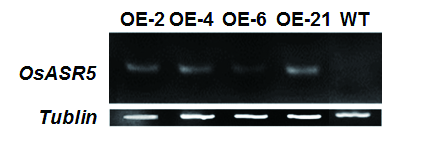


**Fig.S3** Reverse transcript PCR (RT-PCR) analysis of *OsASR5* transcript levels in different *Arabidopsis* transgenic lines. The *Tublin* gene was used as internal controls.


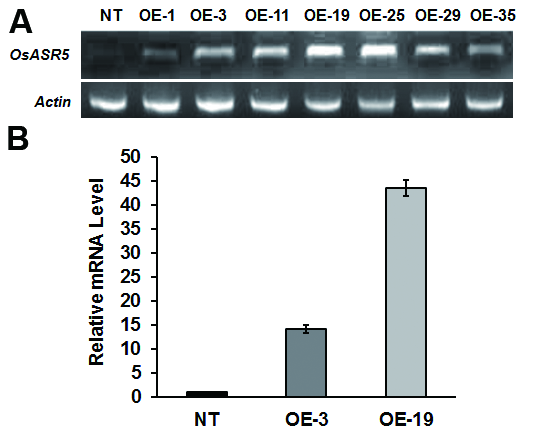


**Fig.S4** Transcription levels of *OsASR5* in *OsASR5* overexpression lines. (A) RT-PCR analysis of *OsASR5* transcript levels in different transgenic lines. The *Actin* gene was used as internal controls. (B) Real-time PCR analysis of *OsASR5* transcript levels in OE-3 and OE-19 transgenic lines. Error bars indicate the SE based on three replicates.


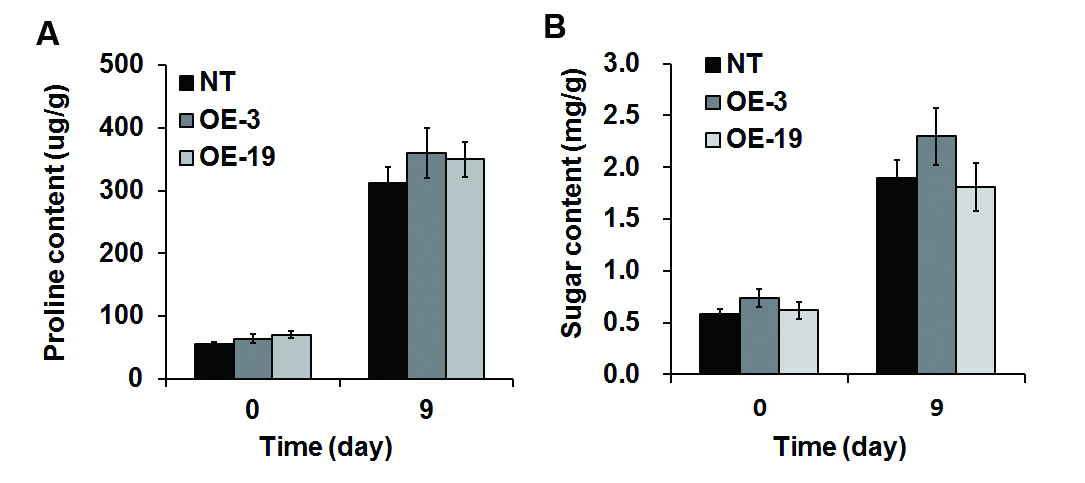


**Fig.S5** Free proline and soluble sugar contents of *OsASR5* overexpression and NT plants under 15% PEG6000 treatment. Free proline (A) and soluble sugar contents (B) were measured in the leaves of three-week-old plants under normal conditions and 15% PEG6000 treatment for 9 day (*n*=3). Error bars indicate the SE based on three replicates.


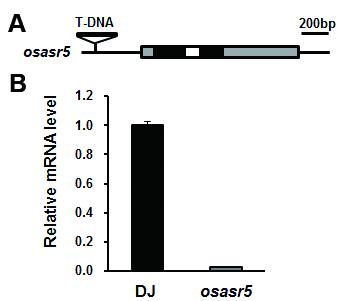


**Fig.S6** Identification of *osasr5* T-DNA insertion mutant. (A) Schematic illustration of the *OsASR5* gene and the location of T-DNA insertion. Exons, intron and untranslated regions are indicated in black, white, and grey, respectively. (B) Real-time PCR analysis of *OsASR5* in *osasr5* mutant. Error bars indicate the SE based on three replicates.


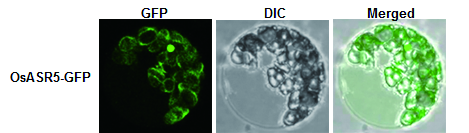


**Fig.S7** Subcellular localization of OsASR5-GFP fusion protein. Protoplasts were isolated from the leaves of transgenic lines harboring a fusion gene of *Pro_OsASR5_*:*OsASR5-*GFP.


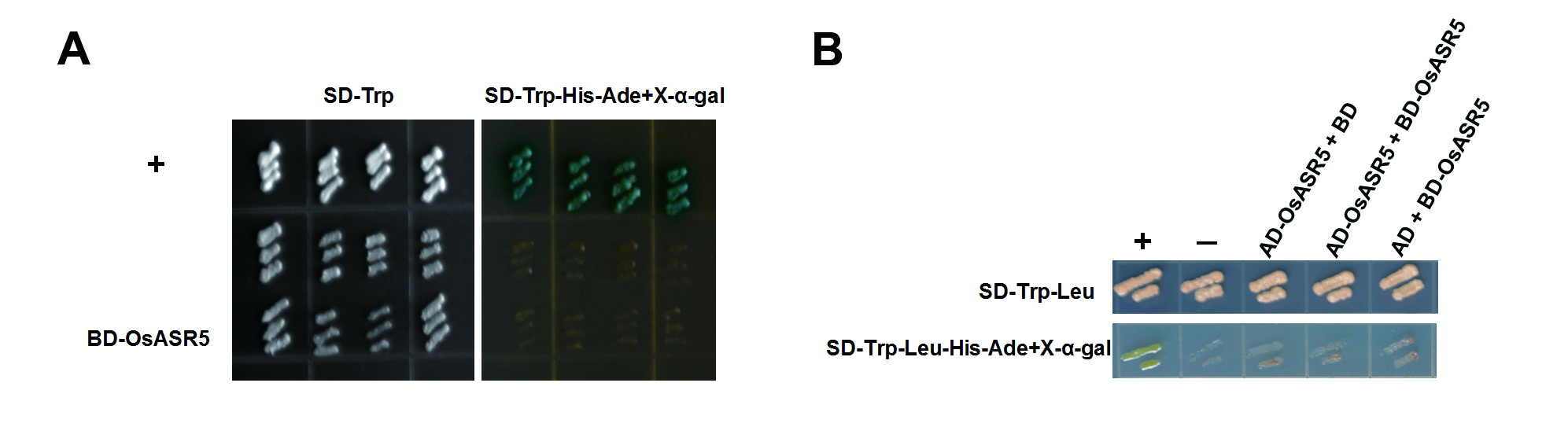


1. **Fig.S8** OsASR5 transcriptional activation and homodimerization analysis. (A) Transactivation assay of OsASR5. Fusion protein of BD-OsASR5 was expressed in yeast strain AH109.The transformed yeast cells were grown on control plate (-Trp).The transactivation activities of OsASR5 were analyzed on selective plate (-Trp/-His/-Ade/ X-α-gal). “+” and “-” indicate the positive and negative control, respectively. (B) Homodimerization analysis of OsASR5. AD-OsASR5 with BD empty, AD-OsASR5 with BD-OsASR5 and AD empty with BD-OsASR5 were co-transformed into yeast strain AH109 and streaked on control plate (-Trp/-Leu) and selective plate (-Trp/-Leu/-Ade/-His/X-α-gal). “+” and “-” indicate the positive and negative control, respectively.
