# Supplementary material for: OsASR5 enhances drought tolerance through a stomatal closure pathway associated with ABA and H2O2 signalling in rice
Source: Plant Biotechnol J. 2016 Nov 11;15(2):183–96. doi: 10.1111/pbi.12601 (PMC5258865; doi:10.1111/pbi.12601)
Supplement: Supplementary file 2 — Table S1 OsASR5 interacting proteins identified in yeast two‐hybrid screening. [file PBI-15-183-s002.docx]

Supplemental Table

**Table S1 OsASR5 interacting proteins identified in yeast two-hybrid screening.**

| **Gene** | **Os ID** | **Locus ID** | **Putative gene Function** |
| --- | --- | --- | --- |
| LHCP | Os09g0346500 | LOC_Os09g17740.1 | Similar to Chlorophyll a-b binding protein chloroplast precursor (LHCII type I CAB) |
| OsSm D1 | Os02g0586500 | LOC_Os02g37430.1 | Similar to Small nuclear ribonucleo protein Sm D1 (snRNP core protein D1) (Sm-D1) (Sm-D autoantigen) |
| Os2OG-Fe (II) oxy | Os01g0830500 | LOC_Os01g61440.1 | 2OG-Fe(II) oxygenase domain containing protein |
| OsMGFP | Os01g0143800 | LOC_Os01g05060.1 | Mitochondrial glycoprotein family protein |
| OsHSP40 | Os01g0556400 | LOC_Os01g37560.3 | Heat shock protein DnaJ family protein |
| OsCS | Os10g0577700 | LOC_Os10g42700.1 | CS domain containing protein |
| OsPCI | Os04g0103200 | LOC_Os04g01290.3 | Proteasome component region PCI domain containing protein |

**Putative gene function was analysed by RGP website (http://rice.plantbiology.msu.edu/).**
