# Supplementary material for: OsASR5 enhances drought tolerance through a stomatal closure pathway associated with ABA and H2O2 signalling in rice
Source: Plant Biotechnol J. 2016 Nov 11;15(2):183–96. doi: 10.1111/pbi.12601 (PMC5258865; doi:10.1111/pbi.12601)
Supplement: Supplementary file 3 — Appendix S1 Supplementary methods. [file PBI-15-183-s001.docx]

## Supplementary methods

## *R**NA isolation and quantitative real-time PCR analysis*

Total RNA was extracted from the leave tissues using Trizol reagent (Takara, Dalian, China), and the first strand cDNA was synthesized using M-MLV Reverse Transcriptase (Promega, Madison, WI, USA) according to the manufacturer’s instructions. Quantitative real-time PCR was performed on the ABI 7500 system (Applied Biosystems, USA). The reaction mixture (20 μL) contained 2 μL of first strand cDNA, 0.5 μM of each of the forward and reverse primers and appropriate amounts of other components as recommended by the manufacturer of SYBR^®^ *Premix Ex Taq*™ II (Takara, Dalian). The rice *Actin* gene was used as the endogenous control to estimate if equal amounts of RNA among the samples were used in the reaction. The respective primers are listed in Supplemental Table S2.

## *Plasmid construction and plant transformation*

To overexpress the *OsASR5* gene, the full-length coding region of *OsASR5* (with locus name LOC_Os011g06720) was inserted into the *Bgl* II and *Pml* I clone sites of the binary vector pCAMBIA1301 under the control of *Cauliflower Mosaic virus* (CaMV35S) promoter. The primers used to generate the *OsASR5* open reading frame (ORF) were listed in Supplemental Table 1. The vector carrying *OsASR5* was transformed into Columbia-ecotype Arabidopsis plants using a simplified floral-dip infiltration method ([Clough and Bent, 1998](file:///E:\李金杰\CAU\20131030%20CAU\Papers\ASR\ASR%20manuscript\2016\Asr5%20manuscript-2016-4-15-PB.docx#_ENREF_9)), and into the *japonica* rice, Nipponbare using *Agrobacterium-*mediated transformation method ([Jeon et al., 2000](file:///E:\李金杰\CAU\20131030%20CAU\Papers\ASR\ASR%20manuscript\2016\Asr5%20manuscript-2016-4-15-PB.docx#_ENREF_28)).

## *S**ubcellular localization of* OsASR5

To investigate the subcellular localization of OsASR5 protein, the full length ORF of *OsASR5* without stop codon was amplified and cloned into the *Pac*I and *Asc*I sites of the binary vector pMDC83, the CaMV35S promoter was replaced by its own promoter. The *Pro_OsASR5_:OsASR5-GFP* construct was introduced into rice as described above. Protoplasts prepared with the leaf tissues of transgenic line with the highest detectable expression of *OsASR5-GFP* were observed under confocal laser scanning microscope (Carl Zeiss, Germany) with an argon laser excitation wavelength (488 nm). Different tissues of transgenic lines were sampled and also observed under confocal laser scanning microscope for analyzing the expression patterns of *OsASR5*.

## *Protein expression and bacterial growth analysis*

The *OsASR5* fragment released by the *BamH* I and *EcoR* I digestions was cloned onto a pGEX-4T-1 expression vector digested with the same enzymes (NEB, USA). The resulting pGST-ASR5 plasmid was transformed into *E.coli* (BL21) for protein expression. The expression of the GST-OsASR5 fusion protein was induced with 0.2 mM isopropylb-D-thiogalactopyranoside for 3 h at 37℃. The cell cultures were serially diluted (1:10, starting OD600nm 1.0) before spotting on LB agar plates supplemented with or without 0.5 M mannitol and incubated at 37℃for 24 h. For growth analysis in liquid medium, 100 µL of cell culture (OD600nm 1.0) was inoculated into 10 mL LB medium supplemented with or without 0.5 M mannitol. Cell growth densities were measured at 600nm.

## *Phy**siological and biochemical indexes assay*

To detect the water loss rate under dehydration conditions, the fourth to sixth-fully expanded leaves of the *Arabidospsis* transgenic and wild type plants grown under normal conditions were detached and weighed immediately as the initial weight. Then the leaf samples were placed on a laboratory bench at room temperature and weighed at the designated time intervals. The water loss rate was calculated on the basis of the initial weight of the leaves ([Xiong et al., 2014](#_ENREF_68)).

To detect the relative water content (RWC) under drought stress conditions, the mid-section of the third-fully expanded leaf blade of *OsASR5* overexpression and NT plants, mutant *osasr5* and DJ were sampled before 15% PEG6000 treatment and after treatment for 3 day, 6 day and 9 day. Fresh weight (FW) of samples was immediately recorded, then samples were soaked for 4 h in distilled water at room temperature under constant light, and the turgid weight was recorded. Total dry weight was recorded after drying these samples at 80°C for 48 h to a constant weight. RWC was calculated according to the standard method ([Barrs and Weatherley, 1962](#_ENREF_4)).

Free proline contents in *OsASR5* overexpression and non-transgenic (NT) lines with or without 15% PEG6000 treatment were determined according to the sulphosalicylic acid method {Bates, 1973 #163}([Bates et al., 1973](#_ENREF_5)). Plant leaves were weighed, frozen in liquid nitrogen, and ground to a fine powder. Acid soluble compounds were extracted by 3% (w/v) sulphosalicylic acid [10 mL/1.5 g FW]. Homogenates were centrifuged for 10 min at 10,000×g. Three-milliliter samples of the supernatants were used to measure free proline content. Total soluble sugars in leaves of *OsASR5* overexpression and NT plants were extracted with 80% ethanol and determined according to the previous method ([Yemm and Willis, 1954](#_ENREF_70)).

Histochemical assay for H_2_O_2_ was conducted by 3,3*φ*-diaminobenzidine (DAB) staining as described previously ([Thordal‐Christensen et al., 1997](#_ENREF_55)). The third-fully expanded leaves of one-month-old *OsASR5* overexpression and NT plants before and after drought treatments were detached and immersed in DAB solution for 8 h at 28°C. The leaf samples were cleared in boiling ethanol (96%) for 20 min and preserved in 50% ethanol for photographing. For quantitative measurement of H_2_O_2_ production, H_2_O_2_ was extracted from leaves and quantified as descried previously ([Uchida et al., 2002](#_ENREF_57)).

## *Tra**nsactivation, Y2H and BiFC assays*

For transactivation assay, the full-length *OsASR5* cDNA was inserted into the pGBKT7 vector (Clontech), pGBKT7:OsASR5 (BD: OsASR5), and transformed into yeast strain AH109. The transactivation experiment was carried out according to the manual of Yeast Protocols Handbook (Clontech). The pGBKT7 was used as a negative control, while the pGBKT7-53 was used as a positive control.

For yeast-two-hybrid （Y2H）screening, the cDNA library (pGADT7: cDNA constructs) was synthesized from mRNA isolated from mixed leaves of UR variety, IRAT109 treated with drought stress. The pGBKT7:OsASR5 (BD: OsASR5) and the pGADT7:cDNA (AD:cDNA) library constructs were co-transformed into yeast strain AH109 and Y2H assay was performed according to the manual of Yeast Protocols Handbook (Clontech).

For bimolecular fluorescence complementation (BiFC) assays, full-length sequences of *OsASR5* and interaction proteins screened by Y2H were inserted into pSPYNE and pSPYCE vectors, respectively. The primer used to generate BiFC constructs was listed in Supplemental Table 1. For transient expression, the BiFC constructs were introduced into *Agrobacterium tumefaciens* strain EHA105, and infiltration of 4-5-week old *N. benthamiana* leaves were performed as described previously ([Li et al., 2013](#_ENREF_38)). The BiFC constructs were also transformed into rice protoplasts isolated from 2-week-old seedlings for in vivo confirmation of interaction, as described previously ([Zhang et al., 2011](#_ENREF_74)). The leaf sections after infiltration 4 days and protoplasts after incubation 24 h were observed via a laser confocal scanning microscopy. The excitation and detec­tion wavelengths for eYFP were 514 nm for excitation and 527 nm for detection.

## *In vitro enzyme aggregation and protection assay*

Protein purifications and digestion of recombinant GST-OsASR5 protein were performed according to the previous method ([Dai et al., 2011](#_ENREF_10)). For enzyme aggregation assay, OsASR5 and BSA (control) were boiled (+) or not (-) at 100˚C for 30 min and then centrifuged at 25,000 *g* for 30 min. The supernatants were used for SDS-PAGE analyzing, as depicted previously ([Dai et al., 2011](#_ENREF_10)).

For enzyme protection assay, a solution of 200 μM freeze-labile lactate dehydrogenase (LDH) was prepared in 25 mM Tris-HCl buffer (pH 7.0) mixed with or without 100 μM BSA, with 50 μM, 100 μM OsASR5. The mixtures were frozen in liquid nitrogen for 30 s and thawed at room temperature for 10 min for various cycles. The enzyme activity of LDH was determined according to the method described previously ([Hsu et al., 2011](#_ENREF_21)). Three independent experiments were performed and all samples were assayed in triplicate.

## *Supplementary references*

Barrs, H. and Weatherley, P. (1962) A re-examination of the relative turgidity technique for estimating water deficits in leaves. *Australian Journal of Biological Sciences* **15**, 413-428.

Bates, L.S., Waldren, R.P. and Teare, I.D. (1973) Rapid determination of free proline for water-stress studies. *Plant and Soil* **39**, 205-207.

Clough, S.J. and Bent, A.F. (1998) Floral dip: a simplified method for Agrobacterium-mediated transformation of Arabidopsis thaliana. *Plant J* **16**, 735-743.

Dai, J.R., Liu, B., Feng, D.R., Liu, H.Y., He, Y.M., Qi, K.B., Wang, H.B. and Wang, J.F. (2011) MpAsr encodes an intrinsically unstructured protein and enhances osmotic tolerance in transgenic Arabidopsis. *Plant cell reports* **30**, 1219-1230.

Hsu, Y.F., Yu, S.C., Yang, C.Y. and Wang, C.S. (2011) Lily ASR protein-conferred cold and freezing resistance in Arabidopsis. *Plant physiology and biochemistry : PPB / Societe francaise de physiologie vegetale* **49**, 937-945.

Jeon, J.S., Lee, S., Jung, K.H., Jun, S.H., Jeong, D.H., Lee, J., Kim, C., Jang, S., Yang, K., Nam, J., An, K., Han, M.J., Sung, R.J., Choi, H.S., Yu, J.H., Choi, J.H., Cho, S.Y., Cha, S.S., Kim, S.I. and An, G. (2000) T-DNA insertional mutagenesis for functional genomics in rice. *Plant J* **22**, 561-570.

Li, J., Pandeya, D., Jo, Y., Liu, W. and Kang, B.-C. (2013) Reduced activity of ATP synthase in mitochondria causes cytoplasmic male sterility in chili pepper. *Planta* **237**, 1097-1109.

Xiong, H., Li, J., Liu, P., Duan, J., Zhao, Y., Guo, X., Li, Y., Zhang, H., Ali, J. and Li, Z. (2014) Overexpression of OsMYB48-1, a novel MYB-related transcription factor, enhances drought and salinity tolerance in rice. *PloS one* **9**, e92913.

Thordal‐Christensen, H., Zhang, Z., Wei, Y. and Collinge, D.B. (1997) Subcellular localization of H2O2 in plants. H2O2 accumulation in papillae and hypersensitive response during the barley—powdery mildew interaction. *The Plant Journal* **11**, 1187-1194.

Uchida, A., Jagendorf, A.T., Hibino, T., Takabe, T. and Takabe, T. (2002) Effects of hydrogen peroxide and nitric oxide on both salt and heat stress tolerance in rice. *Plant Science* **163**, 515-523.

Yemm, E.W. and Willis, A.J. (1954) The estimation of carbohydrates in plant extracts by anthrone. *Biochem. J.* **57**, 508-514.

Zhang, Y., Su, J., Duan, S., Ao, Y., Dai, J., Liu, J., Wang, P., Li, Y., Liu, B., Feng, D., Wang, J. and Wang, H. (2011) A highly efficient rice green tissue protoplast system for transient gene expression and studying light/chloroplast-related processes. *Plant method* **7**, 30.

Table 1 Primer sequences for quantitative real-time PCR and plasmid construction

| **Primer name** | **Gene accession NO.** | **Os ID** | **Forward primer (5' → 3')** | **Reverse primer (5' → 3')** |
| --- | --- | --- | --- | --- |
| **qRT-PCR** |  |  |  |  |
| *Actin* | AK101613 | Os10g0510000 | TTATGGTTGGGATGGGACA | AGCACGGCTTGAATAGCG |
| *OsASR1* | AK063053 | Os01g0959100 | TAAAGATAGTGGAAATGG | AAGTAGTGATAGGGTAGG |
| *OsASR2* | CI477131 | Os01g0959200 | CGCCTTCGCCCTGTATGA | GGTCGATCAGCCGAAGAG |
| *OsASR3* | AK062319 | Os02g0543000 | CGACTAACTCTGTCTGAA | TGAACACGAAGTAAAAGC |
| *OsASR4* | AK061494 | Os04g0423400 | GCTGTGTGAACTTCTTTT | TACTTCTGTTAGCCCCTG |
| *OsASR5* | AK119547 | Os11g0167800 | CACAAGCACATGGAGCAG | CATACGATGACGACGGAG |
| *OsASR6* | AK060804 | Os01g0963600 | GGAGAGAAACCGACCAAC | CTCATAGAGAGCGAAGGC |
| *RAB16A* | [AK121952](http://www.ncbi.nlm.nih.gov/entrez/query.fcgi?cmd=search&db=nucleotide&term=AK121952%5baccn%5d) | [Os11g0454300](http://rapdb.dna.affrc.go.jp/viewer/gbrowse_details/irgsp1?name=Os11g0454300) | CTCGTCTGAGGATGATGGAATG | CTGCTGCTCGCCCTTGTT |
| *RABl6D* | AK109096 | Os11g0453900 | CGGGTAAACAATAAAGTCGTGATG | GCGCACTTACATACAGTGCTACGT |
| *OsNCED4* | NM_00106544 | Os07g0154100 | GATTGCACGGCACCTTCATT | CTCTGTAATTTGATTTTTCACTGGCTAAT |
| *OsNCED5* | AY838901 | Os12g0617400 | GGATGGGCTGAACTTCTTCCAG | CAGCACATTCGTGATGAACCCT |
| *DST* | NM_001058023 | Os03g0786400 | ATCCAAGAAGGCAAGGTCAATC | CACACGAGGAGGAATTGGAAG |
| *Peroxidase24 precursor* | NM_001049620 | Os01g0378100 | GTCTCCAGGACCTCGTCGTC | AAAAGGTTGCAGTGCCCG |
| **plasmid construction** |  |  |  |  |
| *OsASR5-OE* | AK119547 | Os11g0167800 | AGATCTAAGCAAGCAAAGCCACAT | CACGTGGGAGAAAGTCAAGGTTCG A |
| *Pro_OsASR5_*:*OsASR5-*GFP | AK119547 | Os11g0167800 | GTTTAAACTCATTTGATAAGCTTGTATTTTG  TTAATTAAAAGCAAGCAAAGCCACAT | ACTAGTGCTTATTGGCGTCCTACTGG  GGCGCGCCAGCCGAAGAGGTGGTG |
| pGST-ASR5 | AK119547 | Os11g0167800 | GGATCCATGGCGGAGGAGAAGCAC | GAATTCATCAGCCGAAGAGGTGGT |
| YNE-OsASR5 | AK119547 | Os11g0167800 | CGCGGATCCATGGCGGAGGAGAAGCAC | CCGCTCGAGGCCGAAGAGGTGGTG |
| Os2OG-Fe (II) oxy-YCE | AK105801 | Os01g0830500 | CGCGGATCCATGGCCGCCTCCTCCTC | CGGGGTACCCGGCGGTGGAGGAGGAG |
| OsHSP40-YCE | [AK243505.1](http://www.ncbi.nlm.nih.gov/nuccore/116012870) | Os01g0556400 | CGCGGATCCATGGAGGGCAACAAGGA | CGGGGTACCCTGTGCTGTTGCCTCAA |
